# Comparison of Short-Term Associations between PM_2.5_ Components and Mortality across Six Major Cities in South Korea

**DOI:** 10.3390/ijerph16162872

**Published:** 2019-08-11

**Authors:** Si-Eun Yoo, Jin-Soo Park, Soo Hyun Lee, Choong-Hee Park, Chul-Woo Lee, Sang-Bo Lee, Seung Do Yu, Sun-Young Kim, Ho Kim

**Affiliations:** 1 Environmental Health Research Division, National Institute of Environmental Research, Incheon 22689, Korea; 2 Department of Public Health Sciences, Graduate School of Public Health, Seoul National University, Seoul 08826, Korea; 3 Air Quality Research Division, National Institute of Environmental Research, Incheon 22689, Korea; 4 Department of Cancer Control and Population Health, Graduate School of Cancer Science and Policy, National Cancer Center, Goyang, Gyeonngi 10408, Korea

**Keywords:** chemical component, fine particle, mortality, short-term, time-series study

## Abstract

Association between short-term exposure to fine particulate matter (PM_2.5_) and mortality or morbidity varies geographically, and this variation could be due to different chemical composition affected by local sources. However, there have been only a few Asian studies possibly due to limited monitoring data. Using nationwide regulatory monitoring data of PM_2.5_ chemical components in South Korea, we aimed to compare the associations between daily exposure to PM_2.5_ components and mortality across six major cities. We obtained daily 24-h concentrations of PM_2.5_ and 11 PM_2.5_ components measured from 2013 to 2015 at single sites located in residential areas. We used death certificate data to compute the daily counts of nonaccidental, cardiovascular, and respiratory deaths. Using the generalized additive model, we estimated relative risks of daily mortality for an interquartile range increase in each pollutant concentration, while controlling for a longer-term time trend and meteorology. While elemental carbon was consistently associated with nonaccidental mortality across all cities, nickel and vanadium were strongly associated with respiratory or cardiovascular mortality in Busan and Ulsan, two large port cities. Our study shows that PM_2.5_ components responsible for PM_2.5_-associated mortality differed across cities depending on the dominant pollution sources, such as traffic and oil combustion.

## 1. Introduction

Epidemiological studies suggest that the association between short-term exposure to fine particulate matter (PM_2.5_) and mortality or morbidity is possibly due to the association with specific chemical components [1,2,3,4,5]. Ambient PM_2.5_ is a mixture of many chemical components including carbon, trace elements, and inorganic ions. These components originate from different pollution sources, and therefore, identifying specific components responsible for the health effect of PM_2.5_ is of particular interest and expected to provide guidance for policy solutions. For example, elemental carbon (EC) largely emitted from diesel exhaust was found to be strongly associated with mortality and cardiovascular endpoints compared to other components in some epidemiological studies [1,2,6,7,8]. These findings support policy efforts targeting reduction in diesel emissions by developing efficient fuel and installation of emission control devices. 

As emission sources geographically differ, the relative chemical components’ composition of PM_2.5_ varies across areas, and their health effects could also vary accordingly. A review of 41 worldwide studies on PM_2.5_ chemical components and mortality showed substantial regional differences in effect estimates, suggesting higher risks of PM_2.5_ and EC in North America and Europe, subsequently, than in the West Pacific [1]. In addition, some of the chemical components observed with relatively high concentrations in some areas also showed strong associations with mortality or morbidity in the same areas. In the National Morbidity, Mortality, and Air Pollution Study including 69 U.S. communities, the overall mortality effect estimate of PM_10_ decreased substantially when they excluded communities in New York City where nickel (Ni) and vanadium (V) concentrations were particularly high [9,10]. A study performed in two U.S. cities showed higher concentrations of potassium (K), Ni, and EC in Seattle than in Detroit, and found stronger associations of these components with nonaccidental mortality in Seattle [5]. However, there is insufficient evidence to conclude whether the components with higher concentrations and/or higher contribution to PM_2.5_ have greater toxicity compared to other components. 

Different PM_2.5_ chemical compositions and health effects have gained increasing attention in Asian countries, where PM_2.5_ concentrations are high—possibly driven by diverse pollution sources related to rapid urban development—and could affect large populations [11,12]. However, only a few single-city studies have been conducted in Asia, primarily due to the lack of PM_2.5_ chemical component data [11,13,14,15,16,17]. The South Korean government established nationwide PM_2.5_ chemical speciation networks in 2011 in order to identify major sources of PM_2.5_ and monitor the level of chemical component concentrations [18]. The networks include at least one monitoring site in each major city and have sampled daily concentrations of various PM_2.5_ chemical components. This large volume of PM_2.5_ chemical component data provides a unique opportunity to compare their compositions and health effects in multiple cities with diverse pollution sources. 

In this study, we aimed to compare the associations of short-term exposure to PM_2.5_ and 11 PM_2.5_ chemical components to daily mortality across six cities. Specifically, we estimated the association in each of the six major South Korean cities from 2013 through to 2015, and compared the associations across six cities by 12 pollutants and three types of cause-specific mortality. 

## 2. Methods

### 2.1. Study Area

**Figure 1:**
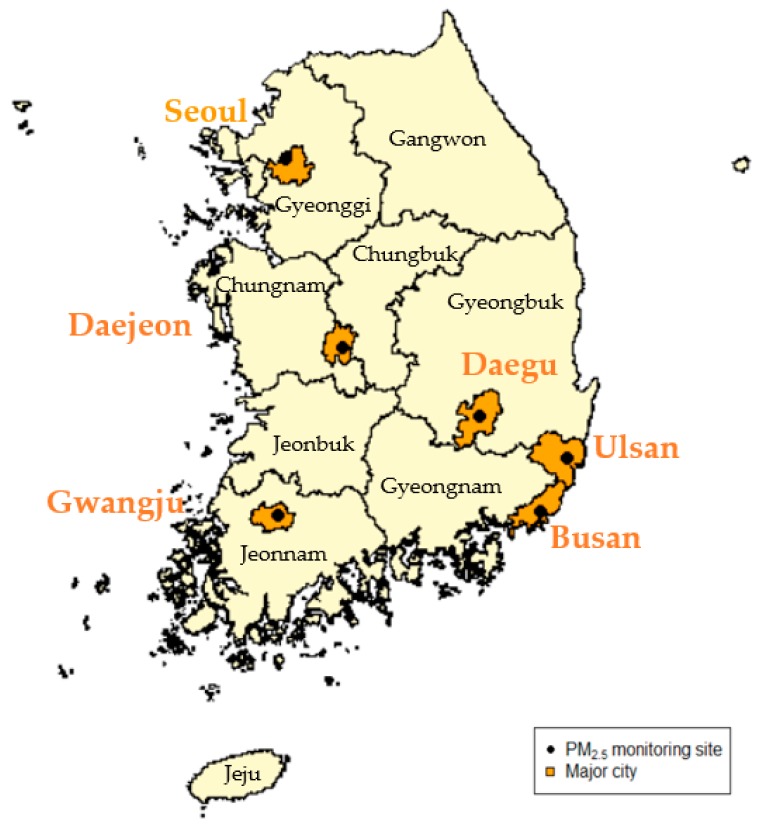
Map of the six major cities (Seoul, Daejeon, Gwangju, Daegu, Ulsan, and Busan) and six PM_2.5_ speciation network monitoring sites in South Korea (nine provinces in black: Gyeonggi, Gangwon, Chungbuk, Chungnam, Gyeongbuk, Gyeongnam, Jeonbuk, Jeonnam, and Jeju).

We used six PM_2.5_ chemical speciation sites located as a single site in each of the six South Korean major cities: Seoul, Daejeon, Gwangju, Daegu, Ulsan, and Busan (Figure 1 and Table S1). South Korea is comprised of seven metropolitan cities and nine provinces. We chose six cities as our study areas where extended time-series data of PM_2.5_ components are available. The average area and population of the six major cities in 2015 were 726.8 km^2^ (range = 501.2, 1060.8) and 3,364,715 people (range = 1,173,534, 10,022,181). Seoul is the capital of South Korea, located in the central part of the Korean peninsula. Busan has grown as the largest seaport in South Korea, and is an economic center of the southeast. Daegu is situated in a basin surrounded by mountains, and has developed into a city with machinery, metal, and textile industries. Gwangju is located in the southwest region, close to expansive agricultural fields. Daejeon is a commercial and industrial city in the central region. Ulsan is another port city located in the southeastern part and well known as its heavy and chemical industries.

### 2.2. PM_2.5_ and PM_2.5_ Components

In South Korea, nation-wide air quality regulatory monitoring networks for criteria pollutants including PM_10_, NO_2_, SO_2_, CO, and ozone were established in 1995. The monitoring networks have expanded to include other pollutants such as PM_2.5_ and PM_2.5_ chemical components, and currently consist of approximately 300 sites throughout the country [19]. The PM_2.5_ chemical speciation networks were established in 2011 and have been operated by the Ministry of Environment. Whereas the reference measurement method for PM_2.5_ is the beta-ray absorption method in South Korea, PM_2.5_ chemical speciation networks quantify PM_2.5_ concentrations using the gravimetric method and specifically focus on the collection of a wide range of PM_2.5_ chemical components. In 2015, 22 PM_2.5_ chemical speciation network sites collected approximately 30 PM_2.5_ components including carbon, inorganic ions, and metallic elements. Twelve sites are located in seven major cities, while 10 sites are in nine provinces. 

We used daily 24-h concentrations of PM_2.5_ and 11 PM_2.5_ components at six chemical speciation network sites in six major cities from 2013 to 2015 obtained from the National Institute Environmental Research. Out of the 12 PM_2.5_ speciation network sites in seven major cities, we excluded three sites that operated for less than three years and one site where heavy metal measurements were missing for two years. Two sites were additionally excluded to maximize samples, as we selected the monitors that ran for a longer period between two sites in each of Seoul and Ulsan. Our study period was restricted to three years from 2013 to 2015 where PM_2.5_ component data are available in all six cities. We used the monitors that operated for the same time period to focus on city-to-city comparisons by avoiding any differences resulting from the samples collected for different time periods rather than at different sites. 

The sampling and lab analyses for PM_2.5_ and PM_2.5_ components were performed following standardized protocols [20]. All six speciation monitoring sites included in this study are operated and maintained directly by the Ministry of Environment. Samples were collected on a daily basis in 2011, and mostly changed to the every third day schedule from September, 2014. Two sites in Daegu and Ulsan adopted the every sixth day schedule. PM_2.5_ samples were collected on 46.2 ± 0.25 mm Teflon filters with a sample flow rate of 16.7 L/min. PM_2.5_ concentrations were quantified as gravitational mass measurements using a microbalance housed in a temperature and humidity controlled chamber. Inorganic ionic compounds were quantified using ion chromatography following the standard methodology for airborne particulate matter. Organic carbon (OC) and EC were collected on pured quarts fibers preheated at 650 °C for 2 hours before sampling, and quantified by the thermal/optical OC/EC analysis. Trace element concentrations were determined by energy dispersive X-ray fluorescence spectrometry. 

Our study focused on EC, OC, nitrate (NO_3_^−^), sulfate (SO_4_^2−^), lead (Pb), Ni, silicon (Si), V, copper (Cu), zinc (Zn), and potassium (K). These 11 PM_2.5_ components have been associated with various health endpoints in previous epidemiological and toxicological studies [1,3,4]. In addition, these components were indicated as surrogates of specific sources, although contributing to more than one sources: EC and OC for mobile emissions of diesel and gasoline engines, respectively; sulfate and nitrate for secondary aerosol formed with fossil and coal combustion and biogenic activity, respectively; Pb for combustion sources including biomass burning and two-stroke engine; Si for wind-blown soil; Ni and V for residual oil burning from ships and power plants; Cu and Zn for industrial emissions such as smelter effluents; and K for biomass burning [5,14,21].

### 2.3. Mortality

Death certificate data from 2013 to 2015 were obtained from the Korea National Statistical Office. Nonaccidental, cardiovascular, and respiratory mortality were defined based on the International Classification of Diseases, 10-th revision code (World Health Organization, 2007): A00-R99, I00-I99, and J00-J99, respectively. Using these certificate data, we computed daily death counts for three types of mortality in each of the six major cities.

### 2.4. Meteorology

We obtained hourly measurements of temperature and relative humidity collected at a meteorology observation site in each of the six major cities from the Korea Meteorological Administration. Then, we computed daily average values of temperature (°C) and relative humidity (%).

### 2.5. Statistical Analysis

We used the generalized additive model (GAM) and fitted Poisson regression to examine short-term associations between the daily concentrations of PM_2.5_ and 11 PM_2.5_ chemical components and daily mortality adjusting for a long-term temporal trend, day of the week, and meteorology. We applied this model to each pair of the 12 pollutants and three types of mortality over all the cities and in each of the six cities. This model allows nonparametric smoothing functions to account for nonlinear relationships of time and temperature with daily death counts. The nonlinear trends of time and temperature were adjusted using thin plate regression splines [22] with 18 (6 per year) and 3 degrees of freedom (df), respectively. To investigate the pattern of delayed effects of air pollution on mortality, we used pollutant concentrations from 0 to 7 days prior to death and estimated eight single-day lag effects (lag 0 to lag 7). In addition, we computed the moving averages for the previous 3–7 days (lag 0–3 and lag 0–7) to explore the impact of exposure over a few days or a week. The associations between daily concentrations of PM_2.5_/PM_2.5_ components and daily mortality were presented as relative risks (RRs) for a city-specific interquartile range (IQR) increase in each pollutant’s concentration with respective 95% confidence intervals (CIs). We applied a city-specific IQR to allow the comparison of RRs across six cities, as the variability of each PM_2.5_ component differs by cities. We also explored whether the PM_2.5_ component with higher contribution to PM_2.5_ shows stronger association with mortality of PM_2.5_ or the corresponding PM_2.5_ component. For this investigation, we looked at the relationship between a ratio of the average concentration of each PM_2.5_ component to PM_2.5_ concentrations and the RR of mortality for the component or PM_2.5_ across six cities. 

We performed two sensitivity analyses to investigate the sensitivity of our results to the modeling choice and data included. First, we changed the degree of adjustment for a long-term temporal trend using smaller or larger dfs (2 and 4 vs. 9 per year) than the primary choice (df = 6), and compared the results to the original findings. Secondly, we expanded our study period to all available years for 2011–2015 and compared to the primary results from 2013 to 2015. The available years varied by six cities: 2011–2015 for Seoul, Gwangju, and Daejeon; 2012–2015 for Daegu and Ulsan; and 2013–2015 for Busan. 

We performed all statistical analyses for PM_2.5_ and 11 PM_2.5_ components at their native scales. Statistical analyses were carried out in SAS version 9.4 (SAS Institute Inc., Cary, NC, USA) and R version 3.5.3 (The R Foundation for Statistical Computing, Vienna, Austria). This study was reviewed and approved by the Institutional Review Board of the Seoul National University (IRB No. E1905/002-001).

## 3. Results

Table 1 and Table S2 summarize the daily concentrations of PM_2.5_ and 11 PM_2.5_ chemical components, daily counts of nonaccidental, cardiovascular, and respiratory deaths, and daily temperature and humidity in each of the six major cities in South Korea, for 3 years from 2013 to 2015. The average daily PM_2.5_ concentrations across the six cities ranged between 22.3 µg/m^3^ in Ulsan and 27.9 µg/m^3^ in Daejeon. The cities located in the central and western regions of South Korea including Seoul, Daejeon, and Gwangju showed high concentrations. The variability was also the highest in Seoul (standard deviation = 18.5 µg/m^3^). In contrast, average concentrations were relatively low in cities of the southeast regions (Busan, Daegu, and Ulsan), with small variability. This regional contrast was generally consistent for the PM_2.5_ components except for OC and some metallic elements. Average daily concentrations of EC, sulfate, and nitrate were higher in Seoul, Daejeon, and Gwangju compared to the three southeastern cities. However, Ni and V were particularly high in Busan, which is the second largest city in South Korea, located on the southeast coast with large ports. Ulsan, another port city, showed the second largest concentrations of Ni and V, although concentrations of PM_2.5_ and most components were the lowest. In addition, OC and Zn showed particularly high concentrations in Daegu compared to all the other cities. Daegu is located 36 km from Gumi, an industrial city for electronics, textiles, fibers, rubber, plastic, and metal products. 

For seasonal variation, PM_2.5_ showed higher concentrations in the winter and lower concentrations in the summer (Figure S1). This seasonal contrast was generally consistent for PM_2.5_ components across cities. However, Ni and V gave a reverse pattern with higher concentrations in the summer and lower concentrations in the winter. EC also showed winter peaks in Seoul but the pattern was inconsistent in Busan. Table 2 provides medians and ranges of correlation coefficients for pairs of PM_2.5_ and PM_2.5_ components across six cities. PM_2.5_ was highly correlated with OC, nitrate, Pb, and K (median r = 0.72–0.76). Although the correlations of PM_2.5_ with Ni and V were low (median r = 0.39–0.50), the correlation between the two components was high (0.75). While some components such as V showed similar correlations with PM_2.5_ across cities, other components such as EC, OC, and nitrate showed large city-to-city variations in their correlations. 

The average number of daily deaths was the highest in Seoul. Out of the 104 nonaccidental deaths per day, 22 and nine people died with cardiovascular and respiratory diseases, respectively. Over the six cities, cardiorespiratory mortality occupies one-fourth to one-third of nonaccidental mortality. Ulsan showed the lowest death counts, possibly because of a large young population compared to those in other cities (average age of 37.4 in Ulsan and 37.5–41.4 in the other five cities) [23]. 

**Figure 2:**
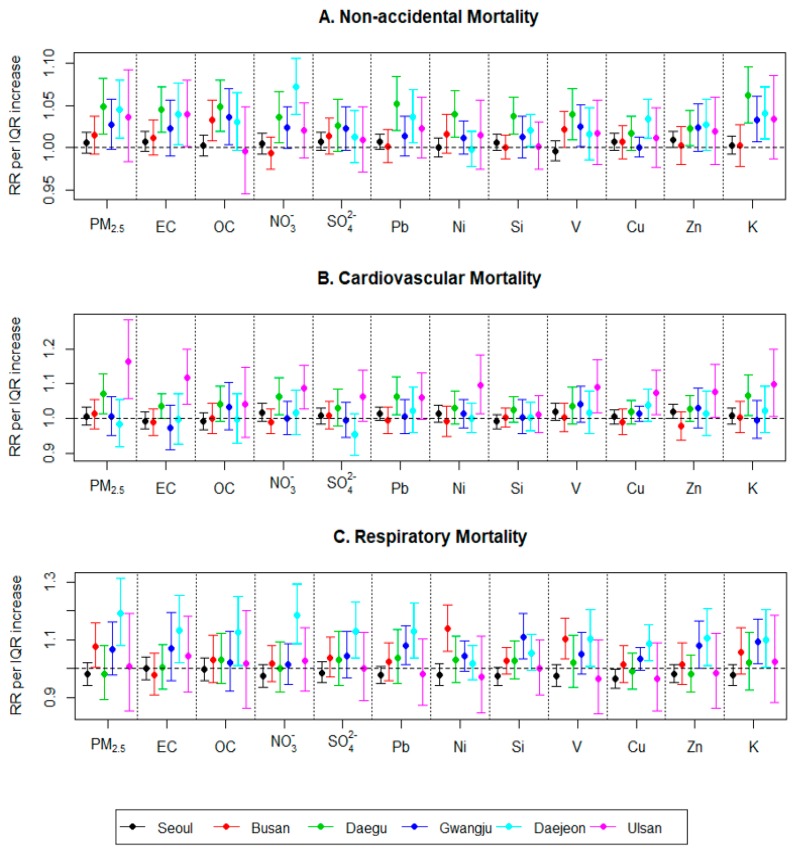
Relative risks (RRs) and 95% confidence intervals of daily nonaccidental, cardiovascular and respiratory mortality for interquartile range increases (IQR) in daily concentrations of PM_2.5_ and 11 PM_2.5_ chemical components across the six South Korean major cities from 2013 to 2015.

Figure 2 shows RRs and 95% CIs of nonaccidental, cardiovascular, and respiratory mortality for IQR increases in PM_2.5_ and each of the 11 PM_2.5_ component concentrations across six cities. We present RRs at lag 1 for Seoul and lag 0 for all the other five cities. Our examination of multiple lags of exposure between 0 and 7 days are shown in Figure S2. RRs of nonaccidental mortality for an IQR increase of PM_2.5_ (16.6–21.7 µg/m^3^) ranged from 1.006 to 1.049 across all six cities, showing significant or marginal associations in three cities (Daegu: RR = 1.049, 95% CI = 1.016–1.083; Gwangju: 1.027, 0.998–1.058; and Daejeon: 1.045, 1.011–1.080). Of the three central and western cities that showed high concentrations of PM_2.5_, Gwangju and Daejeon also showed high RRs, whereas RR was relatively low in Seoul. Although PM_2.5_ concentrations were low in Daegu, RR was higher for nonaccidental mortality compared to all the other cities. This pattern varies by cause-specific mortality. We found the association of PM_2.5_ and cardiovascular mortality in Daegu (RR = 1.070, 95% CI = 1.014–1.128) and Ulsan (1.164, 1.056–1.284), whereas the association was found for respiratory mortality in Busan (1.077, 1.003–1.157) and Daejeon (1.192, 1.081–1.313). 

For 11 PM_2.5_ components and nonaccidental mortality, the patterns of RRs across six cities were similar to those for PM_2.5_. While the overall analysis for all the cities shows that RRs for all pollutants are marginally or significantly positive (Figure S3), city-specific RRs are mostly positive with some variation depending on the components. RRs were generally higher in the central and western cities, Gwangju and Daejeon. Almost all PM_2.5_ components showed significant associations in Daegu. Although there is large variation in the association across PM_2.5_ components, EC was consistently associated with nonaccidental mortality in almost all the cities. There were particularly high and significant RRs for OC and V in Busan; Ni, sulfate, and K in Daegu; and nitrate in Daejeon, compared to those in the other cities. The association patterns for PM_2.5_ components also varied by cause-specific mortality. For cardiovascular mortality, high RRs of nonaccidental mortality found in Daegu mostly remained. However, RRs in Gwangju and Daejeon were close to null, whereas high and significantly positive RRs were found for all components except OC and Si in Ulsan. The patterns for respiratory mortality were generally similar to those for nonaccidental mortality with increased RRs in Gwangju and Daejeon depending on the PM_2.5_ components. In particular, we found increased RRs for Ni and V in Busan. In our analysis of the component contribution to PM_2.5_ and RR, some PM_2.5_ components with higher contributions to PM_2.5_ showed higher RRs for both PM_2.5_ and PM_2.5_ components (Figures S4 and S5). However, this pattern was not clear for all six cities and three types of mortality. In our sensitivity analysis, using greater or smaller df, the results were generally consistent with our primary results (Figure S6). Including a few more years before 2013 depending on the city also gave consistent results (Figure S7).

## 4. Discussion

Using the monitoring data from the PM_2.5_ chemical speciation networks for 3 years in six South Korean major cities, we found some heterogeneity in the short-term association between daily exposure to 11 PM_2.5_ chemical components and daily mortality across major cities where different dominant pollution sources affect different chemical compositions of PM_2.5_. In general, we found higher concentrations of PM_2.5_ components and stronger associations with mortality in cities on the west. However, in Daegu located near large industrial areas where some PM_2.5_ component concentrations such as OC and Zn were notably high, the associations were generally stronger for all components compared to other cities. Ni and V were particularly high in Busan and Ulsan, as large port cities, and gave high mortality effect estimates. Unlike other components, EC showed marginal or significant associations with nonaccidental mortality across all six cities. The patterns also varied by cause-specific mortality. The associations of Ni and V were particularly strong with respiratory mortality in Busan. The association with cardiovascular mortality was strong for all PM_2.5_ components in Ulsan, another large port city. 

To our knowledge, this is the first Asian multicity study that compares the short-term associations between PM_2.5_ components and mortality across major cities using nationwide regulatory monitoring data for PM_2.5_ chemical speciation. All six cities were highly populated with more than one million people, and had relatively high pollution levels with annual average concentrations of PM_2.5_ greater than 22 ug/m^3^. However, the urban environments of these cities vary distinctively, resulting in the variation of dominant sources from human activities such as traffic to heavy industries and large ports. A previous review study also indicates regional differences of source features based on individual source apportionment studies in each region of South Korea [24]. This heterogeneity provides a good opportunity to investigate the similarities and differences in PM_2.5_ component composition and related health effects. Our investigation based on Asian major cities can also help confirm existing findings of the association mostly based on studies in North America and Europe.

Our findings of strong associations for some PM_2.5_ components related to specific pollution sources correspond to scientific understandings from previous studies. Many epidemiological studies suggest that the health effect of PM_2.5_ is attributable to EC and some metallic elements [1,3,25]. In particular, EC consistently shows association with mortality and morbidity, as concluded in a review of 25 cohort studies mostly performed in North America and Europe [26]. As carbon itself is rarely toxic to humans, this indication may suggest various toxic substances related to carbon such as volatile organic compounds [27]. In our study, the association of EC and mortality was consistent throughout all six cities. EC is considered a marker for diesel exhaust resulting from incomplete combustion, and may well represent high traffic congestion in major cities of South Korea as previous source apportionment studies suggested traffic as a major pollution source [24]. Our finding of this association supports the adverse health effect of traffic-related air pollution reported in previous studies [8]. Metallic elements are created mainly in industrial processes such as refining of metals, petroleum, and petrochemicals. Such components can be harmful once accumulated in the body, and have an influence that is proportional to their chemical composition, physical environments, and concentration levels [28,29,30]. In this study, we found high concentrations of Ni and V, with strong associations to respiratory mortality in Busan, established as the largest port in South Korea. A previous study in the U.S. also found that high concentrations of Ni and V in New York City were responsible for the association of PM_2.5_ to mortality in the U.S. [9]. Ni has also been associated to mortality in Xian, China, known as high air pollution concentrations and combustion of fossil fuels such as coal and heavy oil [11]. Toxicological findings also support our findings of Ni toxicity based on in vivo and in vitro studies [31]. 

The variability in 11 PM_2.5_ component concentrations and in their associations with mortality found in our analysis across cities would explain the variability of the association between PM_2.5_ and mortality (0.6 to 4.9% increase of RR for 16.6 to 21.7 ug/m^3^ IQR increase of PM_2.5_), as reported in previous regional studies. For example, Busan and Ulsan with high ratios of Ni and V showed high effect estimates of PM_2.5_ or those components. The different associations between PM_2.5_ and mortality found in some studies in the U.S. and Europe were explained by different chemical composition of PM_2.5_ [3,4,32]. The meta-analysis of WHO regions showed substantial regional variation in the total mortality attributable to PM_2.5_ with RR increase of 0.25 to 2.08%: 1.23% (95% CI: 0.45, 2.01), 0.94% (0.73, 1.16), and 0.25% (0.06, 0.44) in Europe, the U.S., and the Western Pacific, respectively, per 10 ug/m^3^ increase in PM_2.5_ [33]. A recent meta-analysis study including 41 worldwide studies also reported large variability in risk estimates of total, cardiovascular, and respiratory mortality (RR and 95% CI per 10 ug/m^3^ increment in PM_2.5_ = 0.9, 0.7–1.1%; 0.8, 0.4–1.2%; 1.1, and 0.6–1.6%, respectively), with strong associations for EC and K [1]. In region-specific studies, a meta-analysis conducted in six U.S. regions comprising 72 communities, showed that the association between PM_2.5_ and total mortality could be attributed to EC, organic carbon matter, Si, and sodium ions [4]. Another U.S. study, including 75 cities, also showed that total mortality was associated with Si, calcium, and sulfur [3]. In southern Europe, including five countries, magnesium (Mg) and manganese were associated with cardiovascular mortality, whereas sulfate levels were associated with respiratory mortality [32]. For morbidity, a U.S. study including 187 communities and 52 PM_2.5_ components reported the associations of cardiovascular and respiratory hospital admissions with EC, Ni, and V [7]. However, our study shows that high concentrations or proportions of PM_2.5_ components to PM_2.5_ do not necessarily provide strong associations with mortality over all cities. Some cities with high ratios of specific components, such as Ni and V, show high effect estimates of PM_2.5_ or PM_2.5_ components as shown in previous studies [5,9]. Other components and cities did not show consistent patterns. These findings suggest that spatially varying health effects could be due to the contribution of some specific components as well as other factors such as the composition of other components. 

In Asia including South Korea, although there have been few multicity studies, some single-city time-series studies investigated PM_2.5_ components and mortality. However, their findings were inconsistent. In Xian, one study reported the association between nitrate and total mortality [11], whereas another study in the same city showed the association for EC, Pb, Zn, Ni, sulfate, ammonium, sulfur, and chorine, especially during the heating period [15]. In Nagoya, Japan, sulfate was associated with total mortality in elderly people [16]. The two previous studies performed in Seoul showed consistently positive associations between PM_2.5_ and mortality, but findings also varied with respect to contributing components. Heo et al. (2014) [14] collected PM_2.5_ and components at a single sampling site near downtown from 2003 to 2007, and found that an IQR increment in PM_2.5_ was associated with a 2.8% (95% CI: 0.2, 5.4) increase in cardiovascular mortality, which is higher than our RR of 0.6%. For PM_2.5_ components, EC, OC, and Pb showed associations. Based on another sampling campaign at a site located in the eastern part of Seoul from 2008 to 2009, Son et al. (2012) [13] also reported the association of PM_2.5_, but Mg was the only component associated with cardiovascular and total mortality. We also found positive RRs for PM_2.5_, EC, and OC in Seoul, but these were not statistically significant. This discrepancy in study results could be explained by different sampling periods and locations, and/or lab analysis methods [1]. As the PM_2.5_ speciation networks continue to measure PM_2.5_ component concentrations, future studies adding monitoring data for extended years can help elucidate this inconsistency.

We found different associations by cause-specific mortality and lag pattern depending on the city. While there was a delayed pattern in Seoul with maximum effect estimates mostly at lag 1, all the other cities showed higher effect estimates at lag 0. This delayed pattern in Seoul is particularly distinct for respiratory mortality. RRs of respiratory mortality tended to be larger at longer lags and became positive from lag 4 to 6 for all PM_2.5_ components. This pattern resulted in negative associations for most PM_2.5_ components when the identical lag days (1 for Seoul and 0 for all the other cities) were applied to all three types of mortality. Effect estimates were high for cardiovascular mortality in Ulsan, whereas Daejeon and Busan showed high effect estimates for respiratory mortality. Some single-city studies in Denver and Atlanta, U.S., showed the patterns of immediate effects for cardiovascular diseases and delayed effects for respiratory diseases [6,34]. However, our multicity study shows that lag effects and cause-specific mortality effects are relatively homogeneous within a city and distinctively heterogeneous across cities. Prominent distinction in city characteristics including source features across different major cities could result in within-city homogeneity and between-city heterogeneity. The different associations in PM_2.5_ components and mortality across cities could be due to different power or exposure measurement error. However, it is less likely that exposure measurement error driven by using measurements collected from different cities affect our results because we have utilized data with consistent sampling and lab protocols across the six cities.

Our study has several limitations to be addressed in future studies. First, we relied on PM_2.5_ and PM_2.5_ component monitoring data collected at one central monitoring site in each city. These measurements may not represent population levels of exposure, and could result in exposure misclassification and inaccurate health effect estimates particularly for some components known as their large variability at the fine spatial scale. However, when we compared our data to measurements at the second sites in Seoul and Ulsan, excluded in our analysis because of relatively short sampling, PM_2.5_ component concentrations between the two sites were similar in their temporal patterns and were highly correlated (correlation coefficients of 0.96 and 0.76 in Seoul and Ulsan, respectively). These monitoring sites were also located at the top of public office buildings in highly populated areas without particular pollution sources, and can possibly represent population levels of exposure. In addition, this study was designed for assessing population-level relationships between PM_2.5_ components and mortality. Future studies should confirm our finding based on monitoring campaigns that target individual-level exposures to PM_2.5_ components. Lastly, future studies should investigate potential factors that further affect different associations between PM_2.5_ or PM_2.5_ components and health across cities in addition to PM_2.5_ compositions. For example, housing conditions and/or air conditioning could affect residential infiltration and affect city-to-city heterogeneity of the association [35].

## 5. Conclusions

Using regulatory monitoring data for PM_2.5_ chemical components in six South Korean major cities, our study confirms the regional variation in the association between PM_2.5_ components and mortality. This variation is relevant to the dominant pollution sources in each city. While PM_2.5_ and EC showed marginal or significant associations over all cities, a heavily industrialized city showed the associations with most PM_2.5_ components. Large port cities gave strong associations with shipping-related components such as Ni and V. 

## Figures and Tables

**Table 1:** Means and standard deviation of daily concentrations of PM_2.5_ and 11 PM_2.5_ chemical components, and means of daily mortality and weather variables in each of the six South Korean major cities for the period from 2013 to 2015.

City	N	PM_2.5_ (µg/m^3^)	PM_2.5_ Components											Mortality			Meteorology	
			EC (µg/m)	OC _(_µg/m^3^_)_	NO_3_^−^ (µg/m^3^)	SO_4_^2−^ (µg/m^3^)	Pb (ng/m^3^)	Ni (ng/m^3^)	Si (ng/m^3^)	V (ng/m^3^)	Cu (ng/m^3^)	Zn (ng/m^3^)	K (ng/m^3^)	Non- Accidental	CVD	RD	Temp (°C)	RH (%)
Seoul	517	27.1 (18.5)	1.9 (0.9)	4.8 (2.3)	4.7 (5.8)	6.9 (5.7)	28.8 (29.1)	2.2 (2.1)	768.3 (629.4)	5.7 (5.8)	7.9 (7.1)	67.9 (54.1)	349.8 (264.7)	104.2	21.9	9.2	13.2	60.9
Busan	283	23.7 (13.1)	0.8 (0.6)	4.7 (2.3)	2.4 (3.3)	6.1 (4.8)	24.0 (17.3)	3.9 (3.0)	565.1 (532.5)	8.5 (8.9)	7.5 (5.5)	78.4 (49.0)	255.8 (171.6)	50.3	14.0	5.0	15.3	63.5
Daegu	286	23.4 (14.0)	0.8 (0.9)	5.0 (3.1)	2.6 (3.7)	4.8 (3.4)	22.7 (17.5)	2.0 (1.4)	564.1 (488.4)	2.6 (2.4)	7.1 (7.1)	134.9 (138.1)	261.3 (182.5)	31.4	8.7	3.4	15.0	59.3
Gwangju	486	26.0 (16.5)	1.8 (1.1)	4.5 (1.8)	3.4 (4.9)	6.7 (5.6)	28.9 (24.8)	1.9 (2.0)	801.7 (682.0)	4.1 (3.9)	6.1 (8.1)	69.2 (45.0)	352.3 (268.5)	17.3	4.1	2.2	14.4	66.7
Daejeon	437	27.9 (17.0)	2.2 (1.0)	4.7 (1.8)	4.5 (5.9)	6.7 (5.1)	27.5 (24.5)	2.1 (2.3)	744.8 (766.2)	3.6 (3.1)	6.1 (5.1)	64.4 (44.2)	328.8 (251.1)	16.2	3.6	2.0	13.5	71.7
Ulsan	247	22.3 (12.9)	0.7 (0.6)	3.8 (2.1)	1.8 (2.7)	5.6 (4.5)	21.9 (21.2)	2.8 (2.7)	646.2 (676.3)	6.3 (8.0)	5.7 (5.5)	65.1 (50.6)	224.7 (163.5)	11.6	3.3	1.7	14.9	63.1

Abbreviation: EC = elemental carbon; OC = organic carbon; NO_3_^−^ = nitrate; SO_4_^2−^ = sulfate; Pb = lead; Ni = nickel; Si = silicon; V = vanadium; Cu = copper, Zn = zinc, K = potassium; CVD = cardiovascular disease; RD = respiratory disease; Temp = temperature; RH = relative humidity. Six cities ordered by population proportion (city population/national population) in 2015: 19.4, 6.8, 4.8, 2.9, 2.9, and 2.3% for Seoul, Busan, Daegu, Gwangju, Daejeon, and Ulsan, respectively.

**Table 2:** Medians and ranges of correlation coefficients of pairs of PM_2.5_ and 11 PM_2.5_ chemical components across six South Korean major cities from 2013 to 2015.

	EC	OC	NO_3_^−^	SO_4_^2−^	Pb	Ni	Si	V	Cu	Zn	K
PM_2.5_	0.58 (0.41–0.78)	0.72 (0.48–0.83)	0.76 (0.49–0.85)	0.68 (0.57–0.74)	0.72 (0.61–0.81)	0.50 (0.34–0.61)	0.52 (0.41–0.62)	0.39 (0.33–0.48)	0.57 (0.33–0.62)	0.68 (0.56–0.76)	0.76 (0.63–0.81)
EC		0.62 (0.08–0.77)	0.46 (0.25–0.70)	0.34 (0.27–0.40)	0.46 (0.27–0.61)	0.23 (0.10–0.31)	0.19 (0.04–0.42)	0.15 (0.01–0.19)	0.36 (0.21–0.49)	0.42 (0.29–0.58)	0.49 (0.25–0.67)
OC			0.60 (0.39–0.69)	0.37 (0.29–0.53)	0.55 (0.31–0.68)	0.35 (0.22–0.49)	0.38 (0.34–0.45)	0.25 (0.13–0.34)	0.45 (0.28–0.53)	0.54 (0.46–0.65)	0.63 (0.39–0.71)
NO_3_^−^				0.32 (0.20–0.42)	0.59 (0.41–0.72)	0.24 (0.08–0.42)	0.27 (0.13–0.34)	0.07 (−0.14–0.28)	0.52 (0.26–0.56)	0.50 (0.35–0.59)	0.62 (0.52–0.67)
SO_4_^2−^					0.50 (0.42–0.57)	0.46 (0.24–0.52)	0.37 (0.32–0.44)	0.48 (0.36–0.53)	0.32 (0.15–0.44)	0.48 (0.27–0.55)	0.46 (0.44–0.49)
Pb						0.46 (0.30–0.62)	0.47 (0.36–0.51)	0.35 (0.11–0.54)	0.70 (0.46–0.85)	0.72 (0.58–0.87)	0.72 (0.57–0.81)
Ni							0.36 (0.20–0.45)	0.75 (0.46–0.91)	0.58 (0.40–0.72)	0.61 (0.41–0.70)	0.38 (0.24–0.48)
Si								0.44 (0.23–0.50)	0.39 (0.25–0.47)	0.48 (0.34–0.58)	0.67 (0.62–0.74)
V									0.43 (−0.02–0.57)	0.49 (0.11–0.59)	0.32 (−0.01–0.45)
Cu										0.76 (0.46–0.88)	0.56 (0.42–0.66)
Zn											0.65 (0.52–0.74)

## Supplementary Materials

The following are available online at https://www.mdpi.com/1660-4601/16/16/2872/s1, Table S1. Average areas and populations of six South Korean major cities in 2015; Table S2. Interquartile range concentrations of PM_2.5_ and 11 PM_2.5_ components in six South Korean major cities from 2013 to 2015; Figure S1. Time-series plots of daily log-transformed concentrations of PM_2.5_, elemental carbon (EC), nickel (Ni), organic carbon (OC), copper (Cu), and vanadium (V), in the two largest South Korean cities (left: Seoul, right: Busan) from 2013 to 2015 (red lines for smoothed lines based on locally weighted scatterplot smoothing); Figure S2. Relative risks (RRs) and 95% confidence intervals of daily mortality for interquartile range increases in daily concentrations of PM_2.5_ and 11 PM_2.5_ components across seven single-day lags (lag 0 to lag 7) and two multiday lags (lag 0–3 and lag 0–7), in each of the six South Korean major cities from 2013 to 2015; Figure S3. Relative risks (RRs) and 95% confidence intervals of daily nonaccidental mortality for interquartile range increases in daily concentrations of PM_2.5_ and 11 PM_2.5_ chemical components over all six South Korean major cities from 2013 to 2015; Figure S4. Relative risks (RR)s and 95% confidence intervals (CIs) of mortality for PM_2.5_ components against the ratios of PM_2.5_ component concentrations to PM_2.5_ concentrations across six South Korean major cities from 2013 to 2015 (ratio = 100 X PM_2.5_ component concentration/PM_2.5_ concentration; for PM_2.5_, RRs were plotted against concentrations); Figure S5. Relative risks (RR)s and 95% confidence intervals (CIs) of nonaccidental mortality for PM_2.5_ against the ratios of PM_2.5_ component concentrations to PM_2.5_ concentrations across six South Korean major cities from 2013 to 2015 (ratio = 100 X PM_2.5_ component concentration/PM_2.5_ concentration; for PM_2.5_, RRs were plotted against concentrations); Figure S6. Relative risks (RRs) and 95% confidence intervals of nonaccidental mortality for 11 PM_2.5_ components by different degrees of freedom (df) used for temporal trend adjustment (our primary model with df 6 per year); Figure S7. Relative risks (RRs) and 95% confidence intervals of daily mortality for interquartile range increases in daily concentrations of PM_2.5_ and 11 PM_2.5_ components for all available years between 2011 and 2015.
