# Supplementary material for: Comparison of Short-Term Associations between PM2.5 Components and Mortality across Six Major Cities in South Korea
Source: Int J Environ Res Public Health. 2019 Aug 11;16(16):2872. doi: 10.3390/ijerph16162872 (PMC6720204; doi:10.3390/ijerph16162872)
Supplement: Supplementary file 1 [file ijerph-16-02872-s001.pdf]

Supplementary Materials: Comparison of Short-Term Associations between $PM_{2.5}$ Components and Mortality across Six Major Cities in South Korea

Table S1. Average areas and populations of six South Korean major cities in 2015.

City	Area (km^2)	Population
Seoul	605.3	10,022,181
Busan	769.8	3,513,777
Daegu	883.6	2,487,829
Gwangju	501.2	1,472,199
Daejeon	540.8	1,518,775
Ulsan	1,060.8	1,173,534

Table S2. Interquartile range concentrations of $PM_{2.5}$ and 11 $PM_{2.5}$ components in six South Korean major cities from 2013 to 2015

City	$PM_{2.5}$	EC	OC	NO_3^-	SO_4^{2-}	Pb	Ni	Si	V	Cu	Zn	K
Seoul	21.7	1.1	2.8	6.2	6.5	23.5	2.3	627.7	6.3	6.9	55.6	286.3
Busan	16.6	0.7	3.2	3.1	5.6	19.4	3.8	443.3	9.7	6.0	61.8	214.1
Daegu	18.1	0.7	3.5	3.7	4.2	21.2	1.6	414.9	2.6	5.9	124.6	238.2
Gwangju	19.3	1.4	2.5	4.3	5.9	25.0	1.6	698.1	3.9	3.8	51.5	294.0
Daejeon	21.4	1.4	2.4	6.0	6.0	26.7	1.9	586.5	3.5	4.4	51.7	287.7
Ulsan	18.1	0.5	2.9	2.1	4.7	21.3	2.8	504.4	7.5	5.3	56.7	204.5

A. $PM_{2.5}$

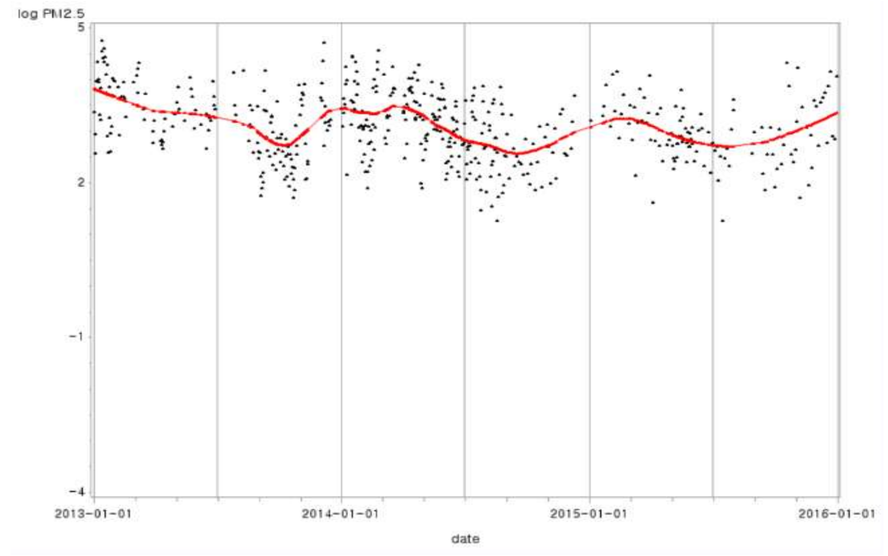
log PM2.5
5
2
-1
-4
2013-01-01
2014-01-01
2015-01-01
2016-01-01
date

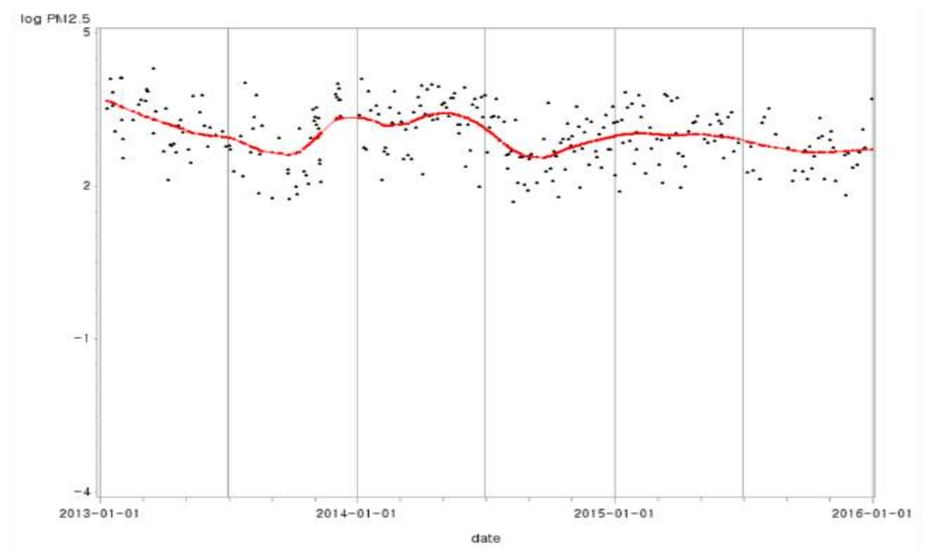
log PM2.5
5
2
-1
-4
2013-01-01
2014-01-01
2015-01-01
2016-01-01
date

B. EC

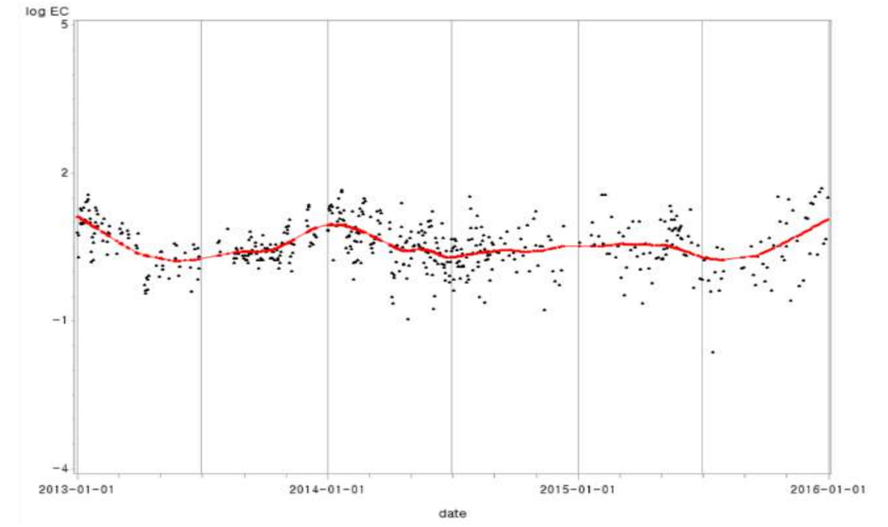
log EC
5
2
-1
-4
2013-01-01
2014-01-01
2015-01-01
2016-01-01
date

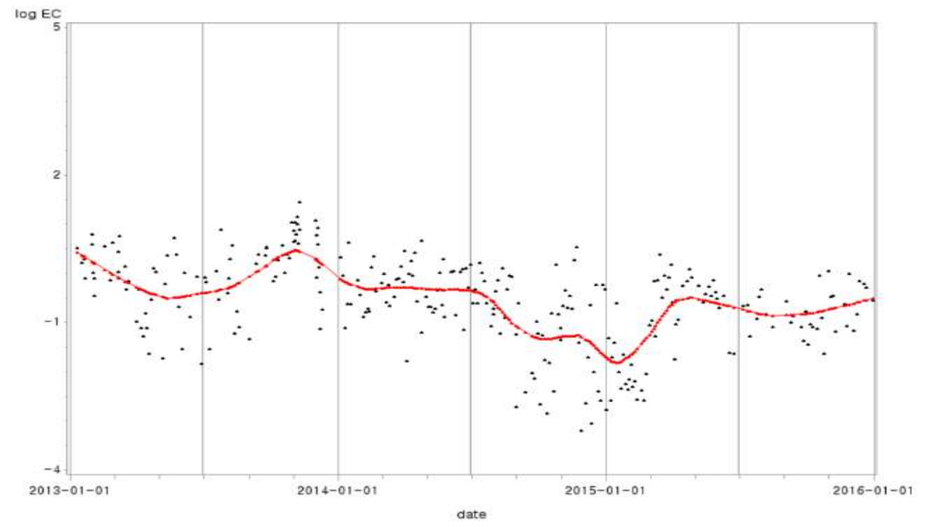
log EC
5
2
-1
-4
2013-01-01
2014-01-01
2015-01-01
2016-01-01
date

C. Ni	
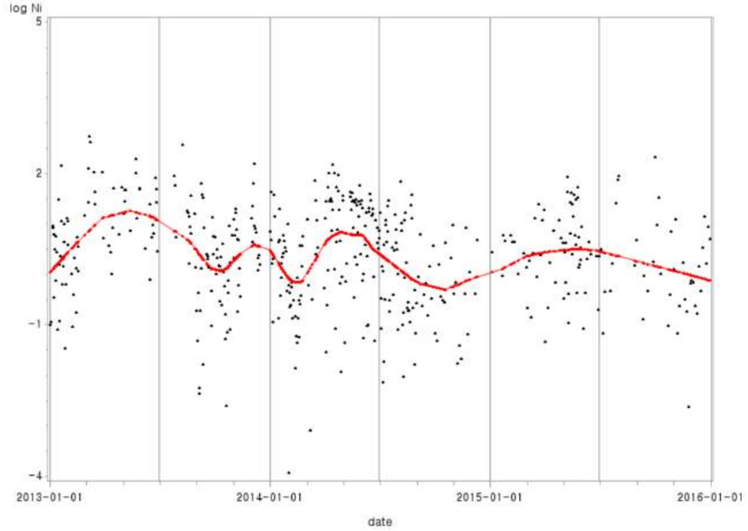log Ni<br>5<br>2<br>-1<br>-4<br>2013-01-01<br>2014-01-01<br>2015-01-01<br>2016-01-01<br>date	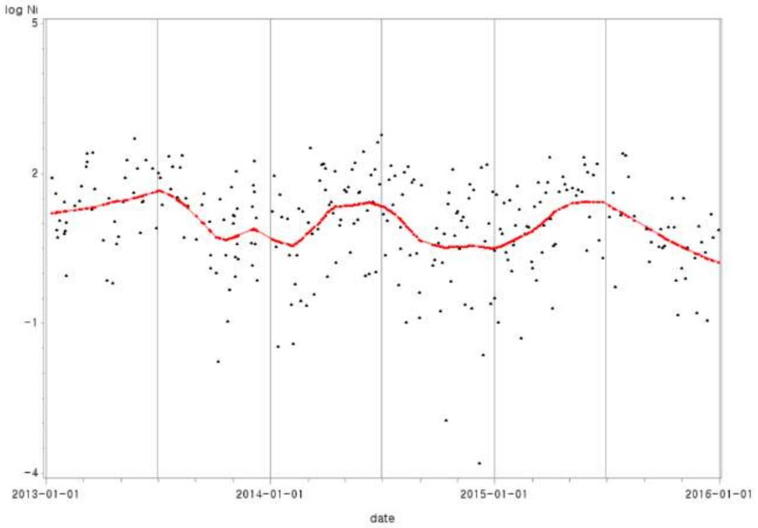log Ni<br>5<br>2<br>-1<br>-4<br>2013-01-01<br>2014-01-01<br>2015-01-01<br>2016-01-01<br>date
D. OC	
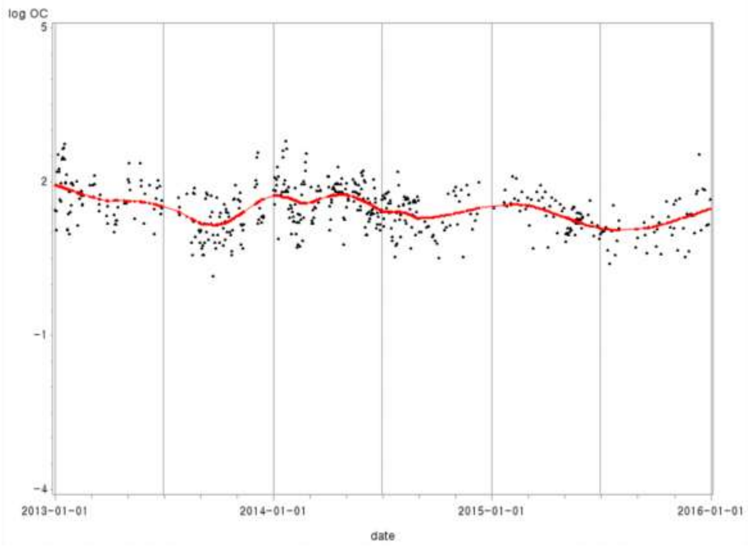log OC<br>5<br>2<br>-1<br>-4<br>2013-01-01<br>2014-01-01<br>2015-01-01<br>2016-01-01<br>date	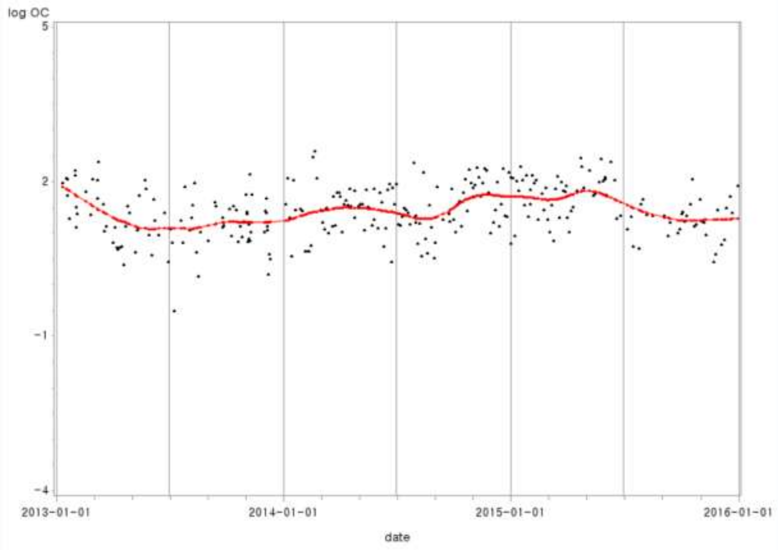log OC<br>5<br>2<br>-1<br>-4<br>2013-01-01<br>2014-01-01<br>2015-01-01<br>2016-01-01<br>date

E. Cu

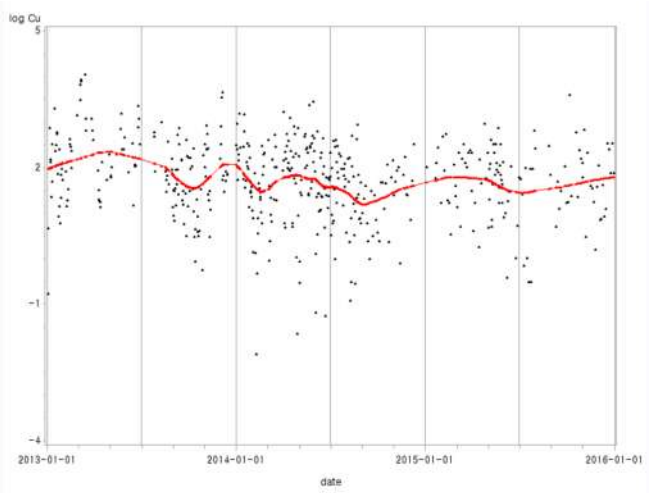

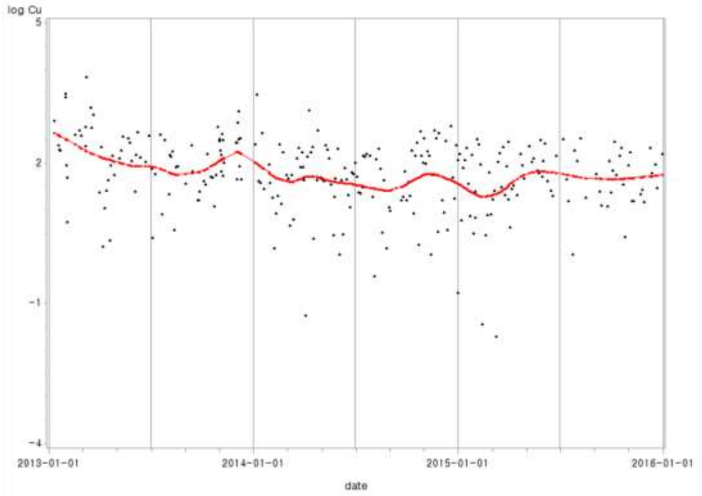

F. V

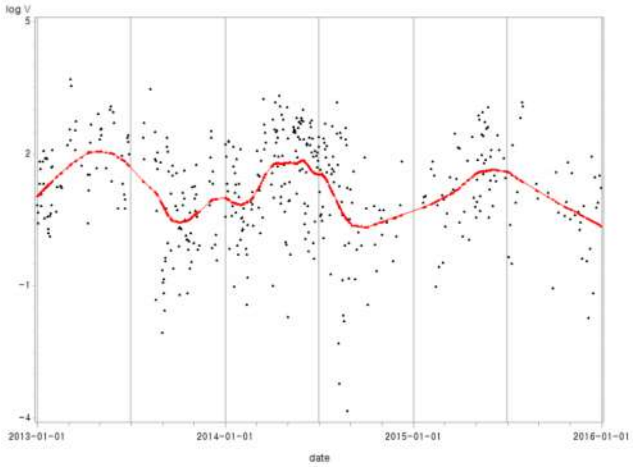

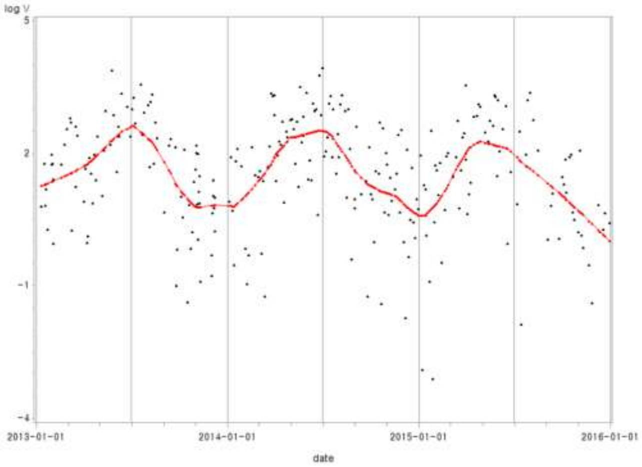

Figure S1. Time-series plots of daily log-transformed concentrations of $PM_{2.5}$, elemental carbon (EC), nickel (Ni), organic carbon (OC), copper (Cu), and vanadium (V), in the two largest South Korean cities (left: Seoul, right: Busan) from 2013 to 2015 (red lines for smoothed lines based on locally weighted scatterplot smoothing).

A. Non-accidental mortality

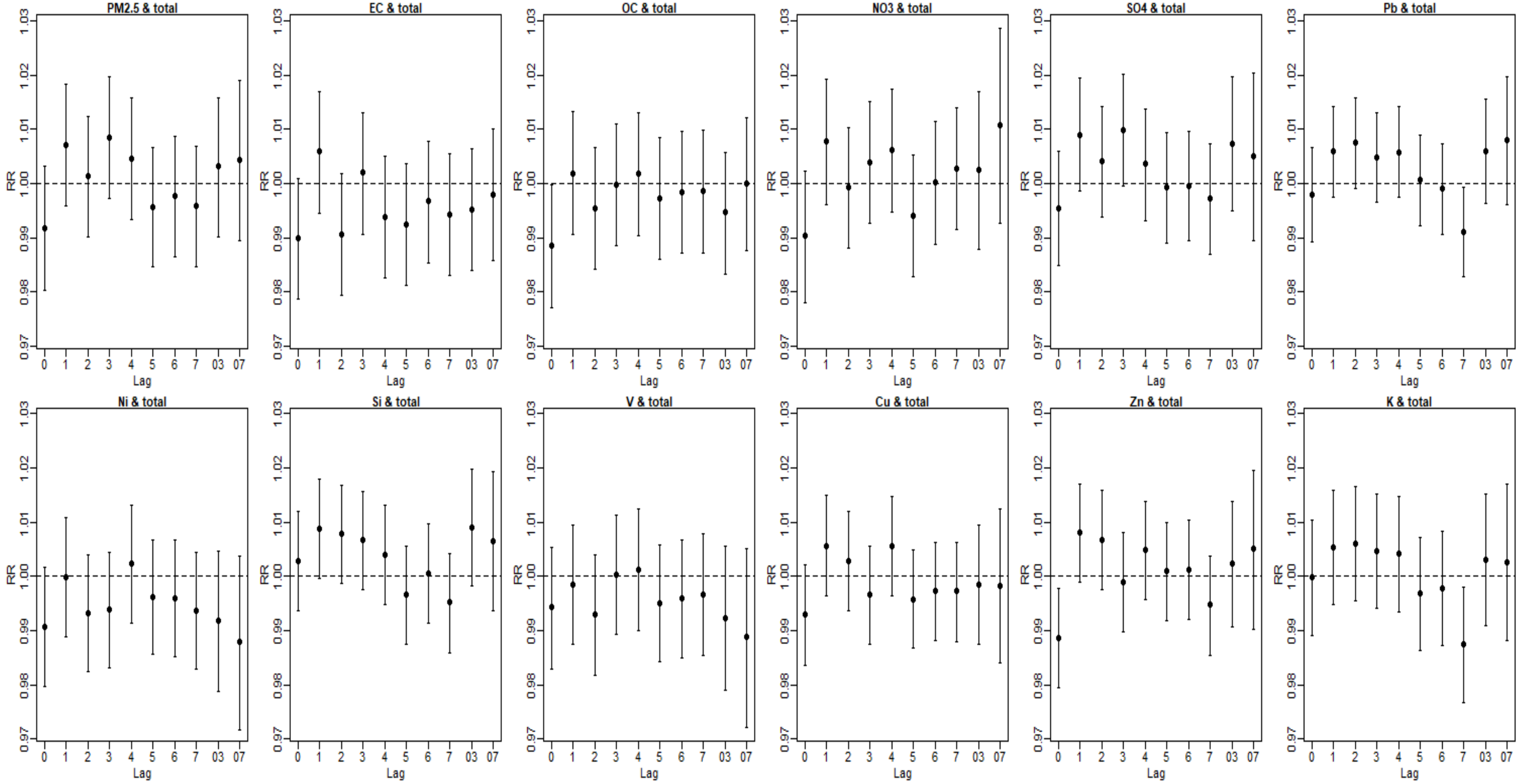

Seoul

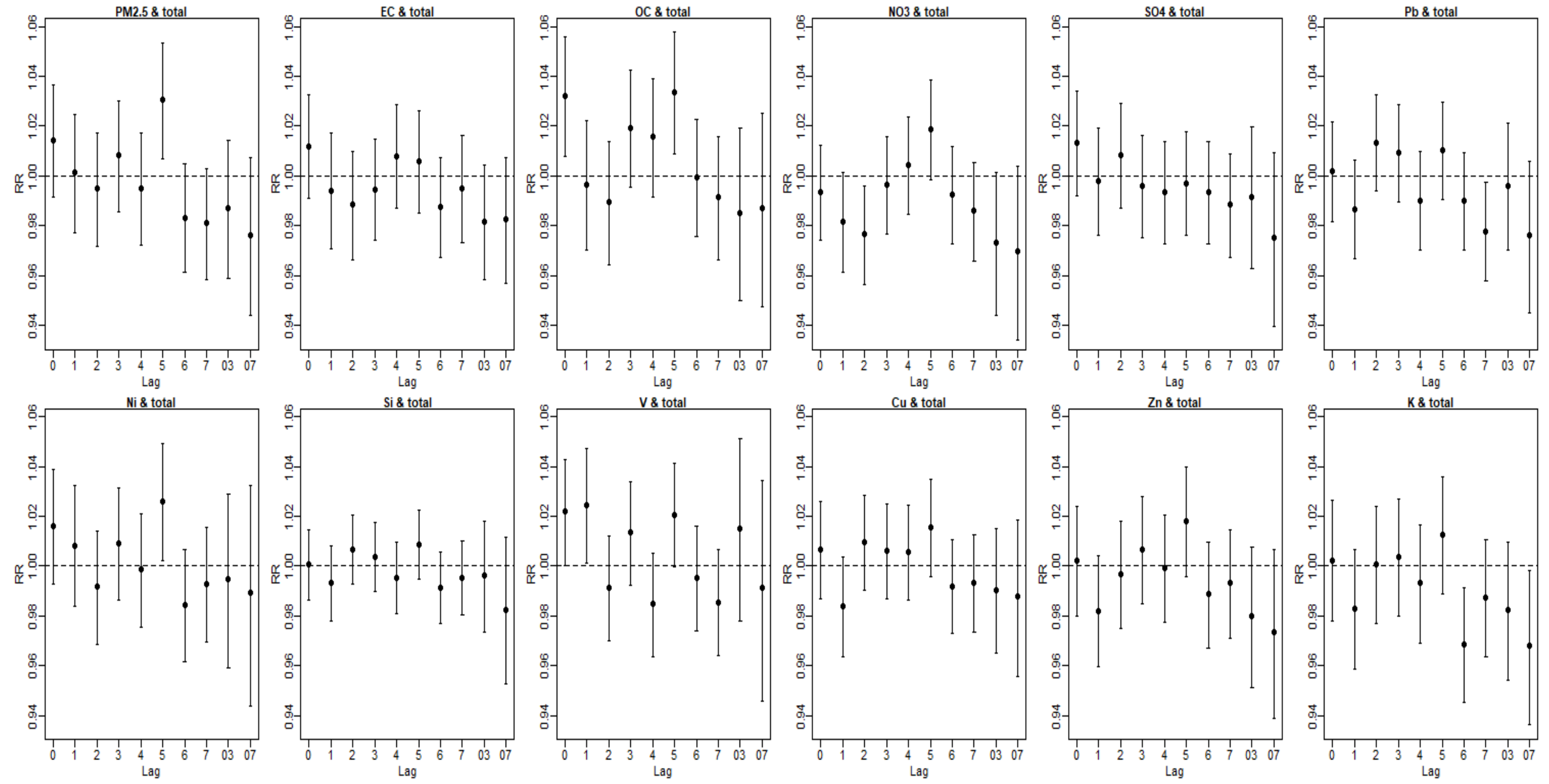
PM2.5 & total
EC & total
OC & total
NO3 & total
SO4 & total
Pb & total
Ni & total
Si & total
V & total
Cu & total
Zn & total
K & total
RR
Lag

Busan

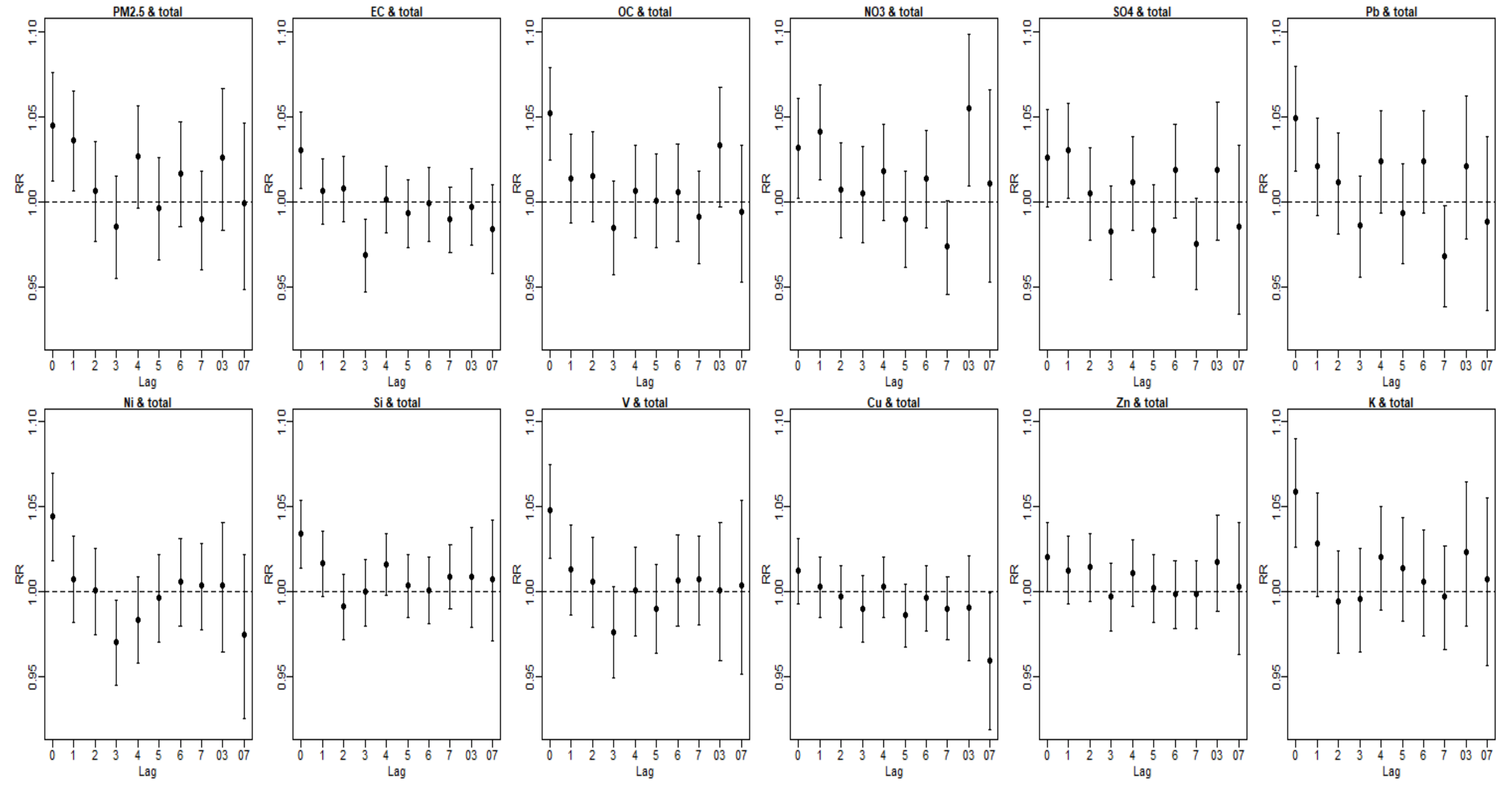
PM2.5 & total
EC & total
OC & total
NO3 & total
SO4 & total
Pb & total
Ni & total
Si & total
V & total
Cu & total
Zn & total
K & total
RR
Lag

Daegu

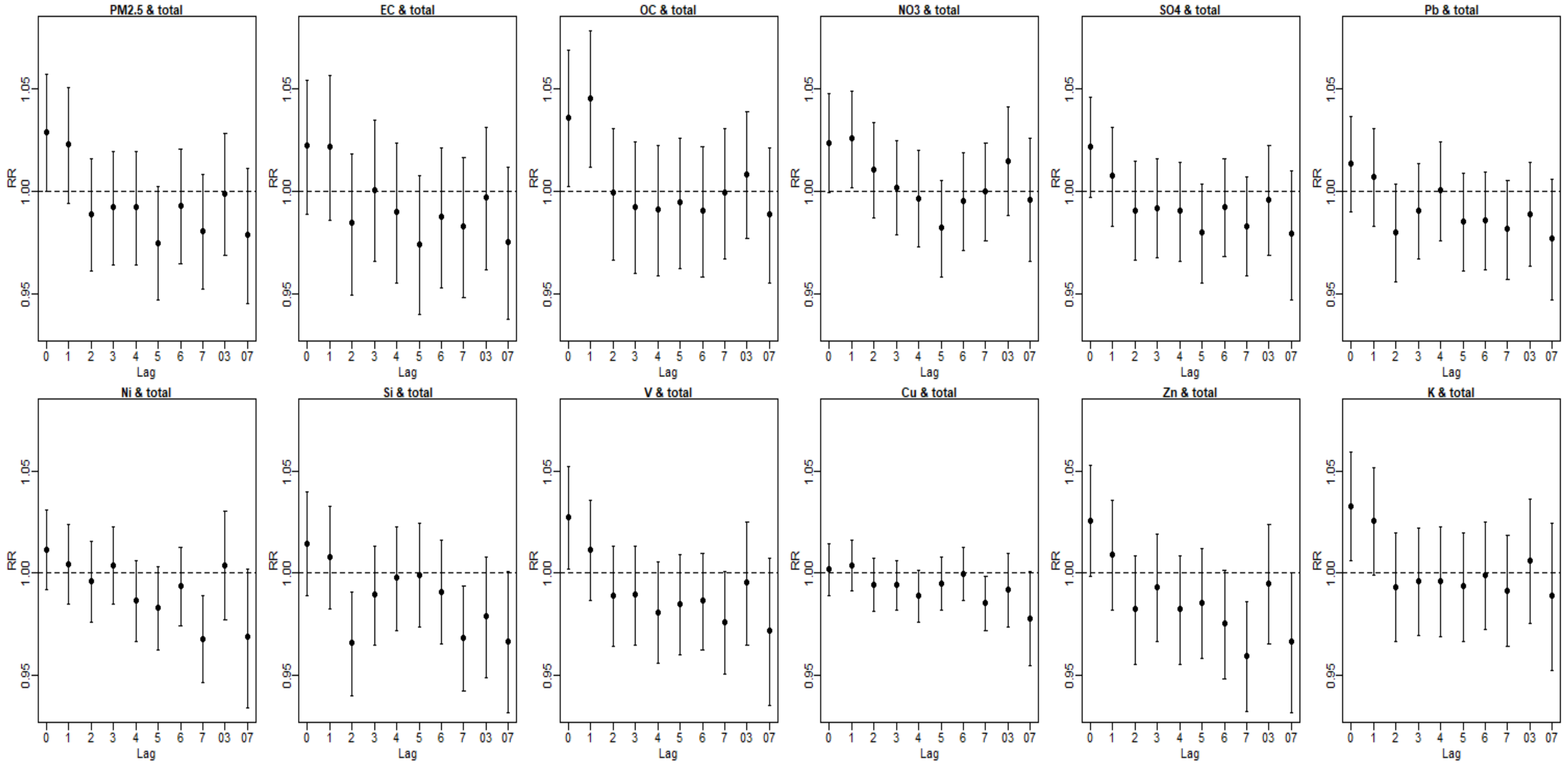
PM2.5 & total
EC & total
OC & total
NO3 & total
SO4 & total
Pb & total
Ni & total
Si & total
V & total
Cu & total
Zn & total
K & total
RR
0.95
1.00
1.05
0 1 2 3 4 5 6 7 03 07
Lag

Gwangju

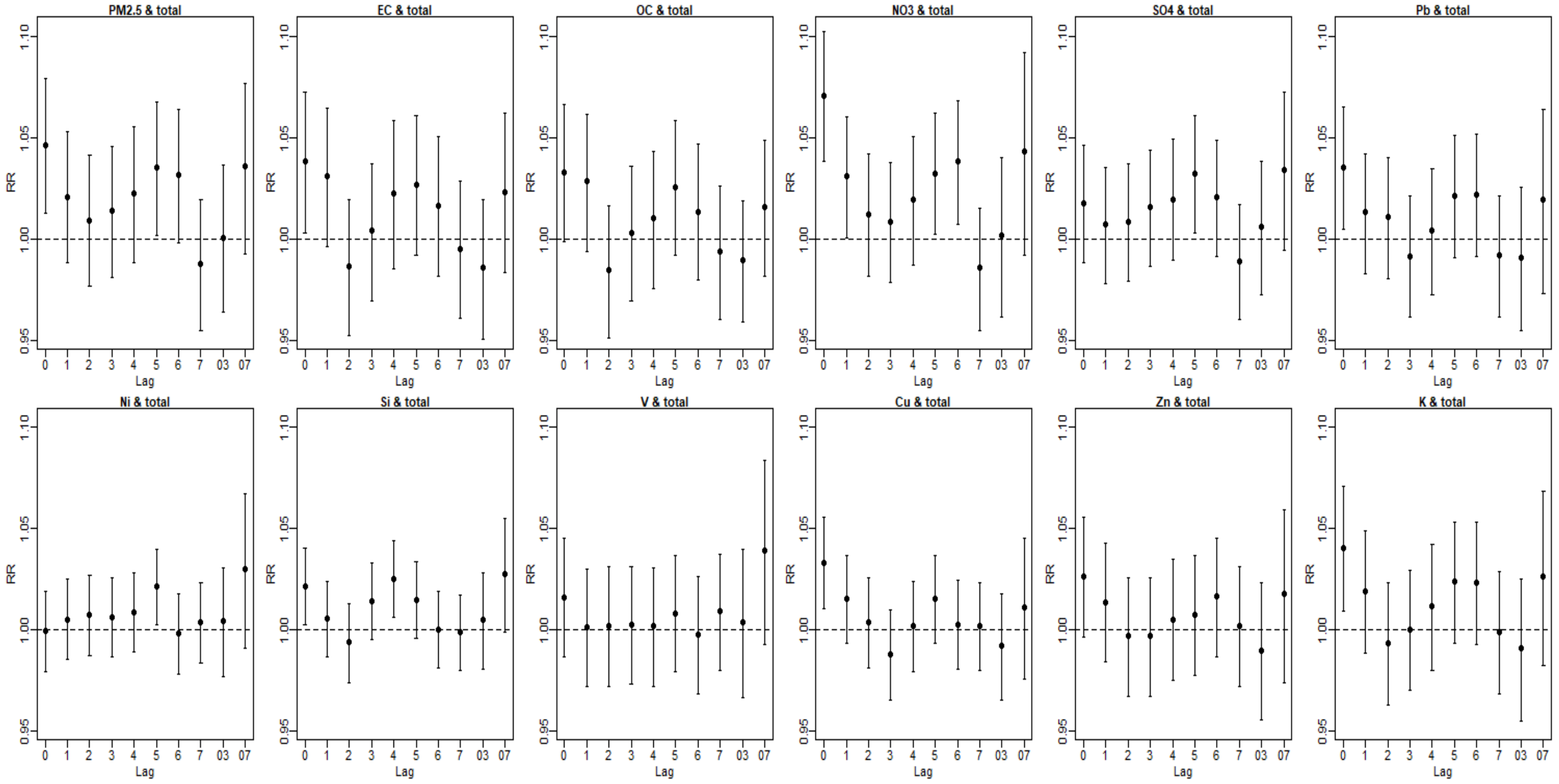
PM2.5 & total
EC & total
OC & total
NO3 & total
SO4 & total
Pb & total
Ni & total
Si & total
V & total
Cu & total
Zn & total
K & total
RR
Lag

Daejeon

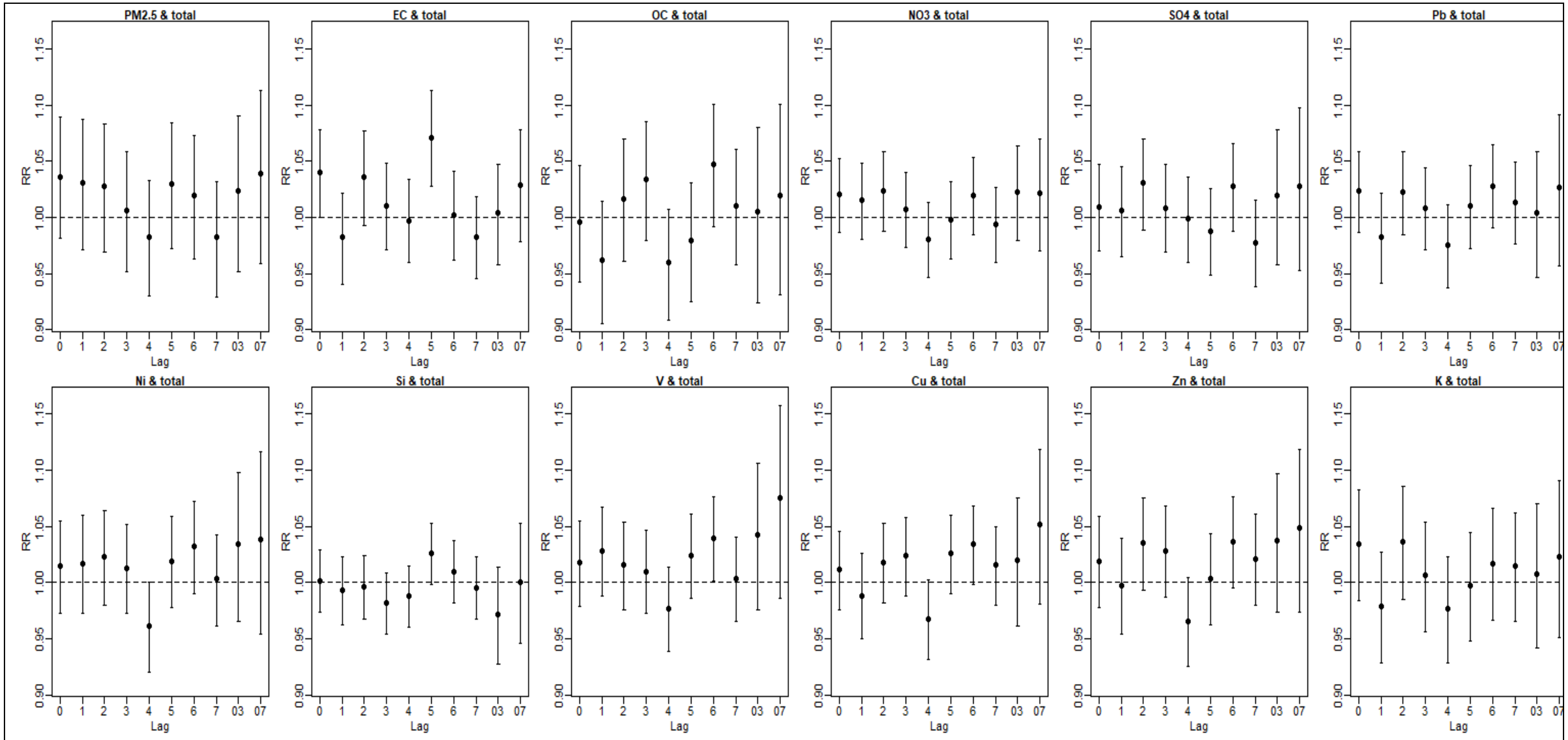

PM2.5 & total
EC & total
OC & total
NO3 & total
SO4 & total
Pb & total
Ni & total
Si & total
V & total
Cu & total
Zn & total
K & total
RR
Lag
0.90
0.95
1.00
1.05
1.10
1.15
0 1 2 3 4 5 6 7 03 07

Ulsan

B. Cardiovascular mortality

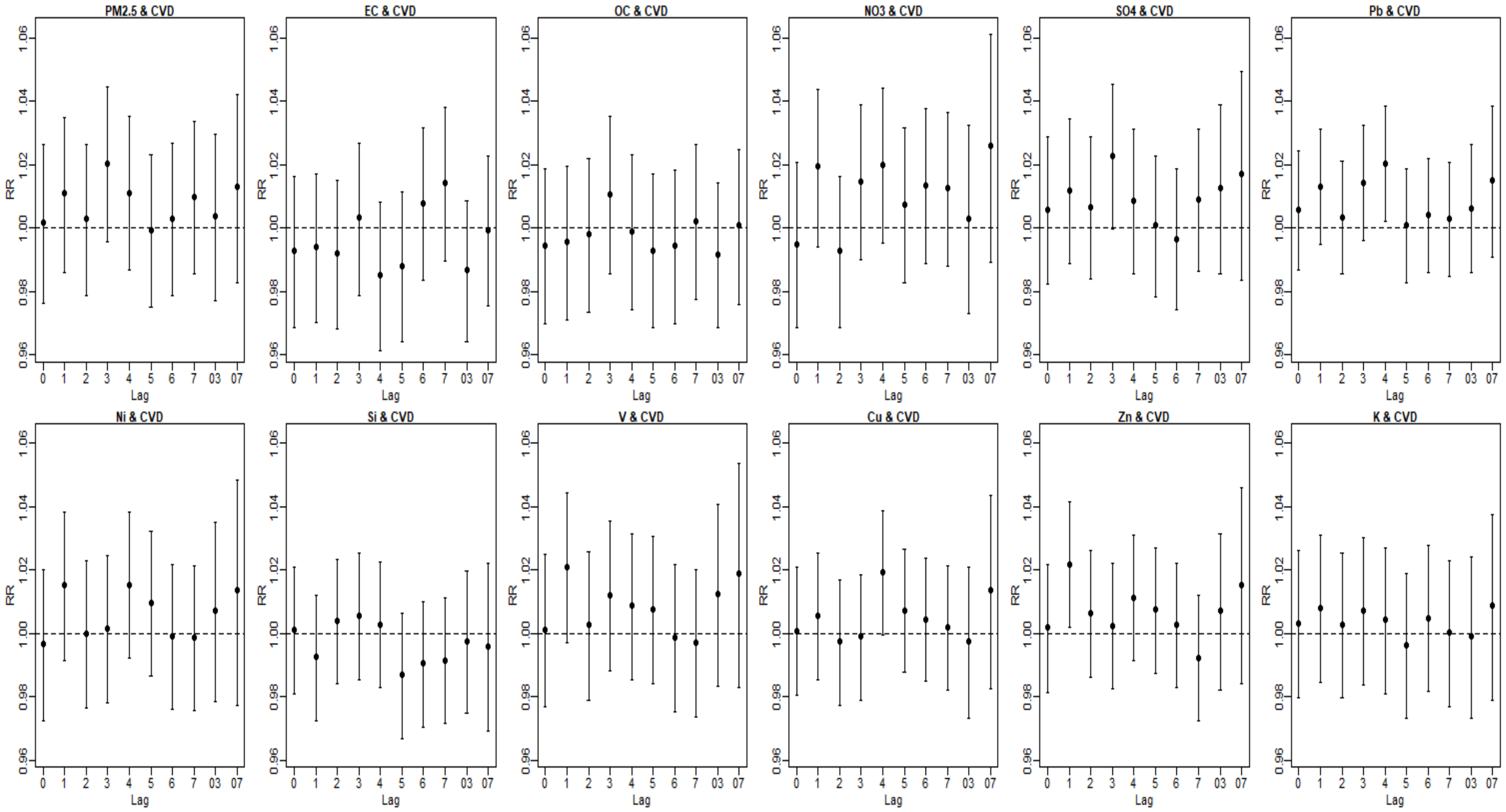

Seoul

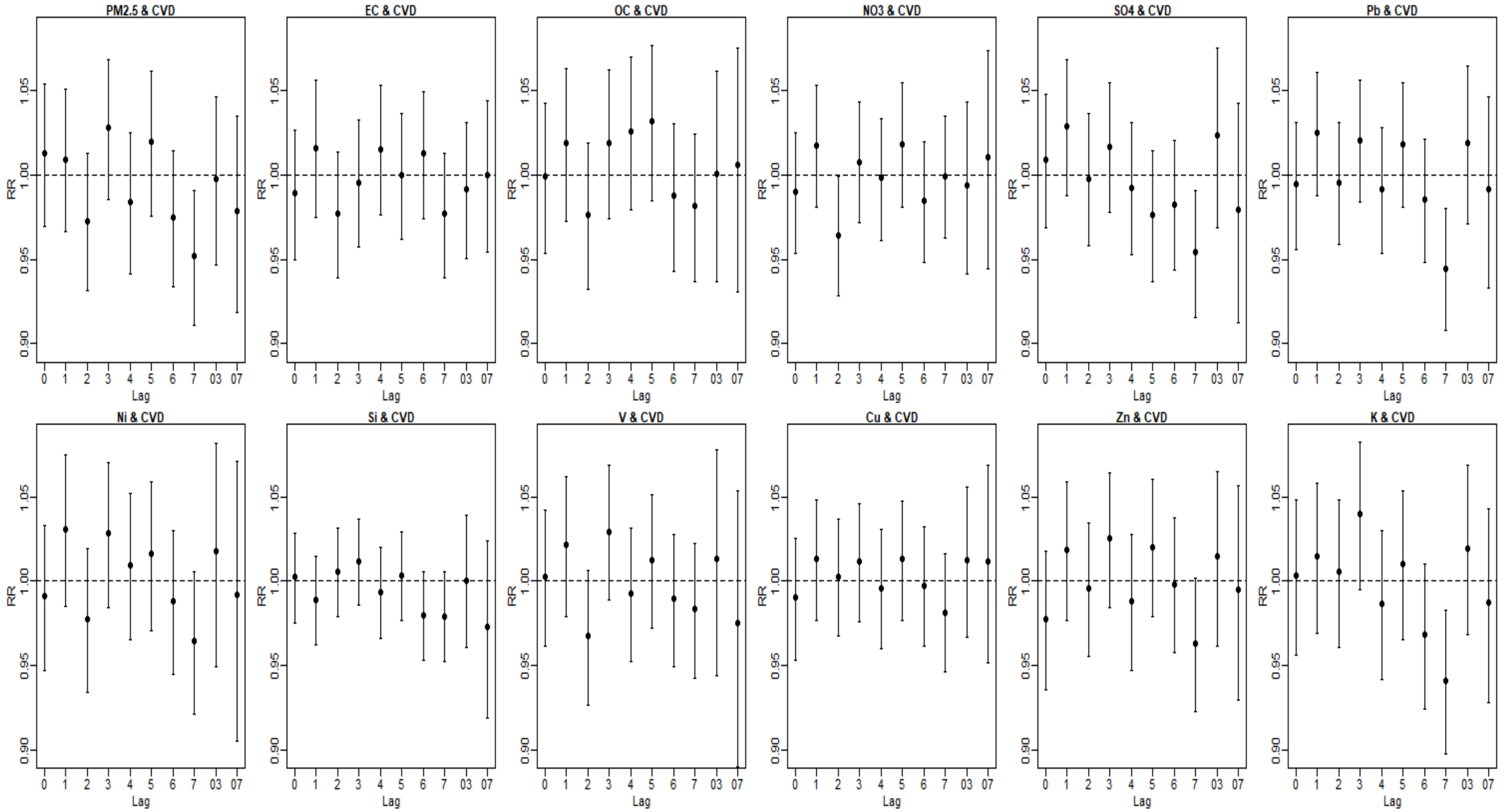
PM2.5 & CVD
EC & CVD
OC & CVD
NO3 & CVD
SO4 & CVD
Pb & CVD
Ni & CVD
Si & CVD
V & CVD
Cu & CVD
Zn & CVD
K & CVD
RR
0.90 0.95 1.00 1.05
Lag
0 1 2 3 4 5 6 7 03 07

Busan

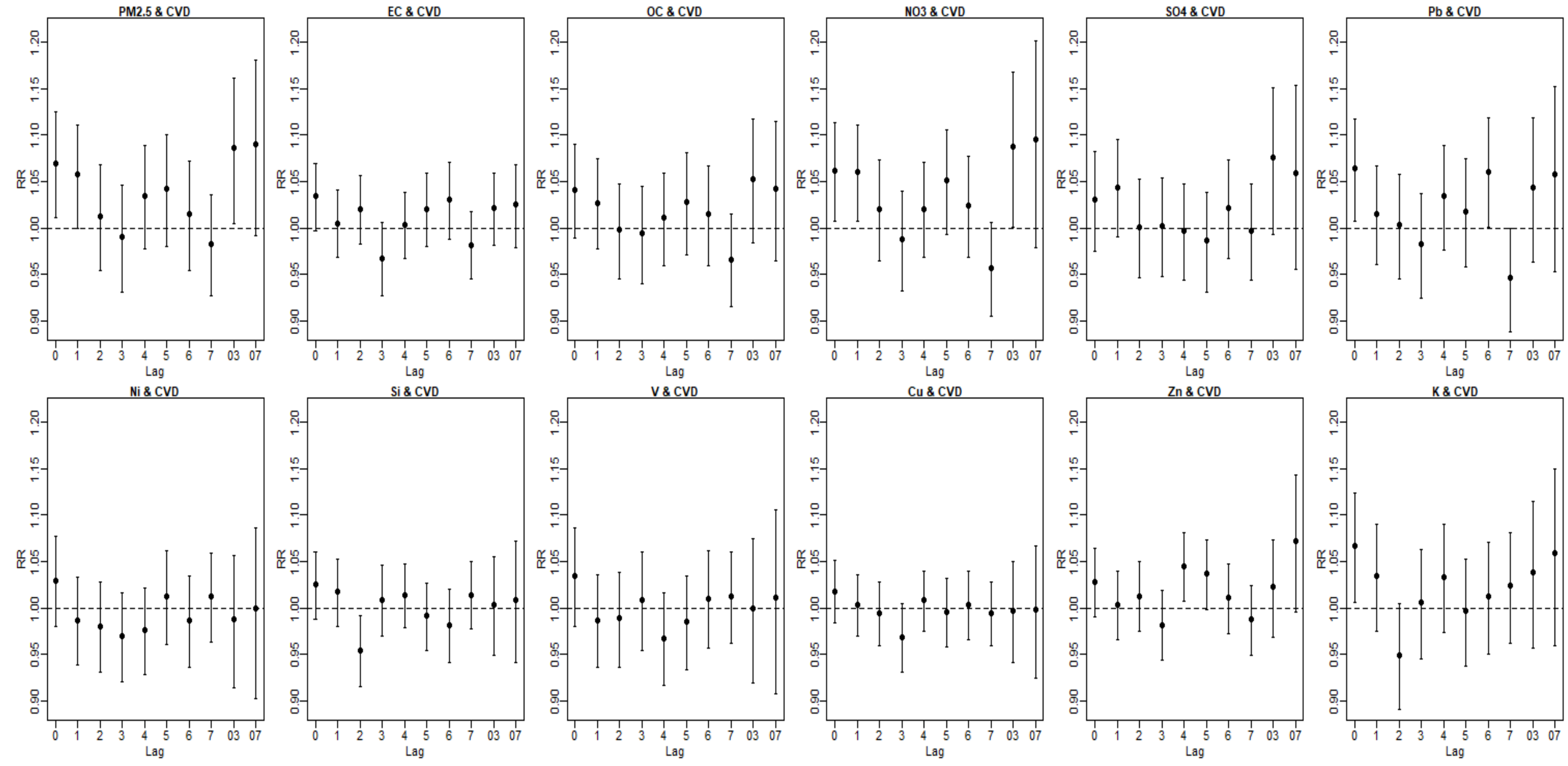
PM2.5 & CVD
EC & CVD
OC & CVD
NO3 & CVD
SO4 & CVD
Pb & CVD
Ni & CVD
Si & CVD
V & CVD
Cu & CVD
Zn & CVD
K & CVD
RR
Lag

Daegu

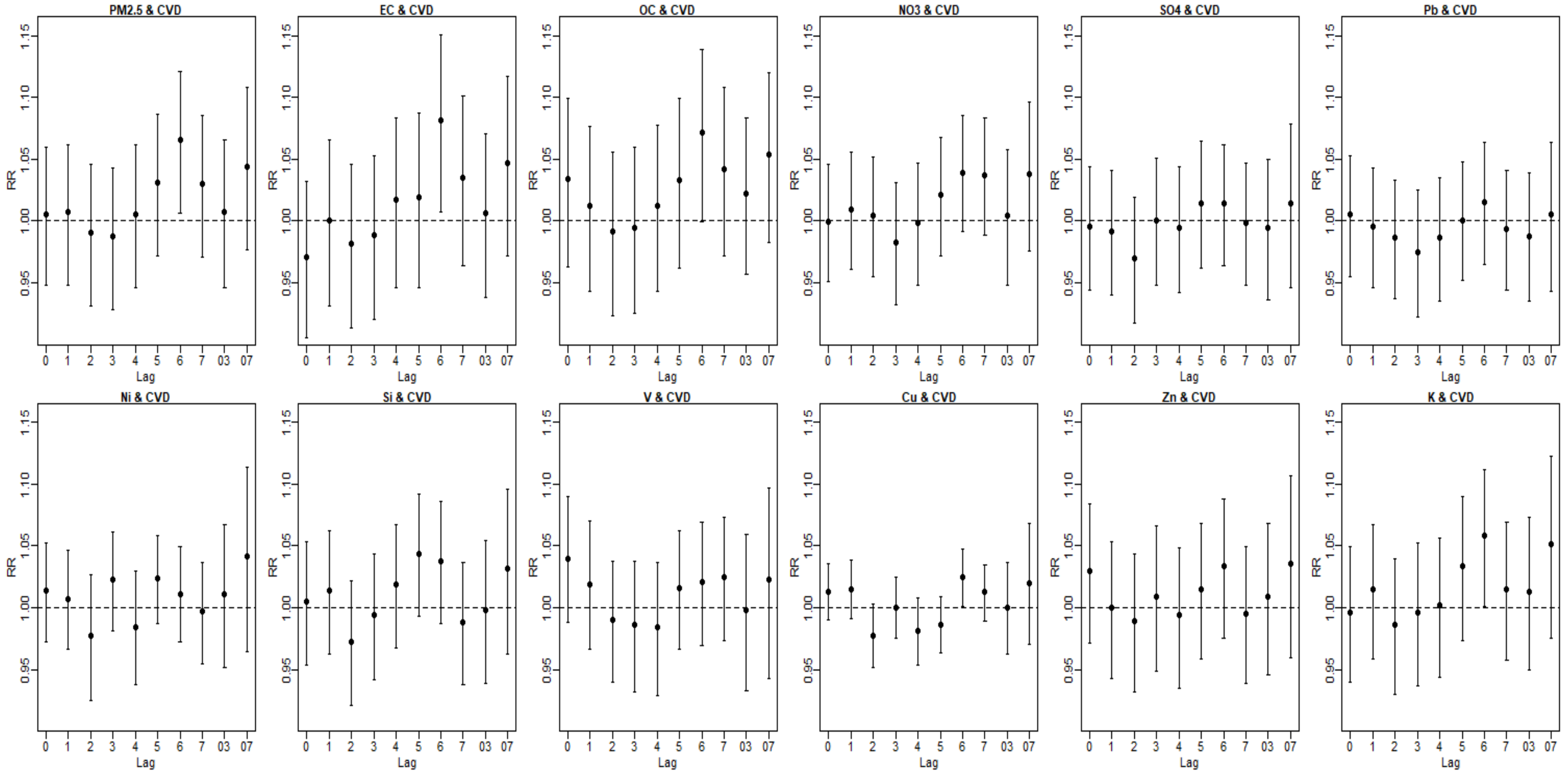
PM2.5 & CVD
EC & CVD
OC & CVD
NO3 & CVD
SO4 & CVD
Pb & CVD
Ni & CVD
Si & CVD
V & CVD
Cu & CVD
Zn & CVD
K & CVD
RR
Lag
0.95
1.00
1.05
1.10
1.15
0 1 2 3 4 5 6 7 03 07

Gwangju

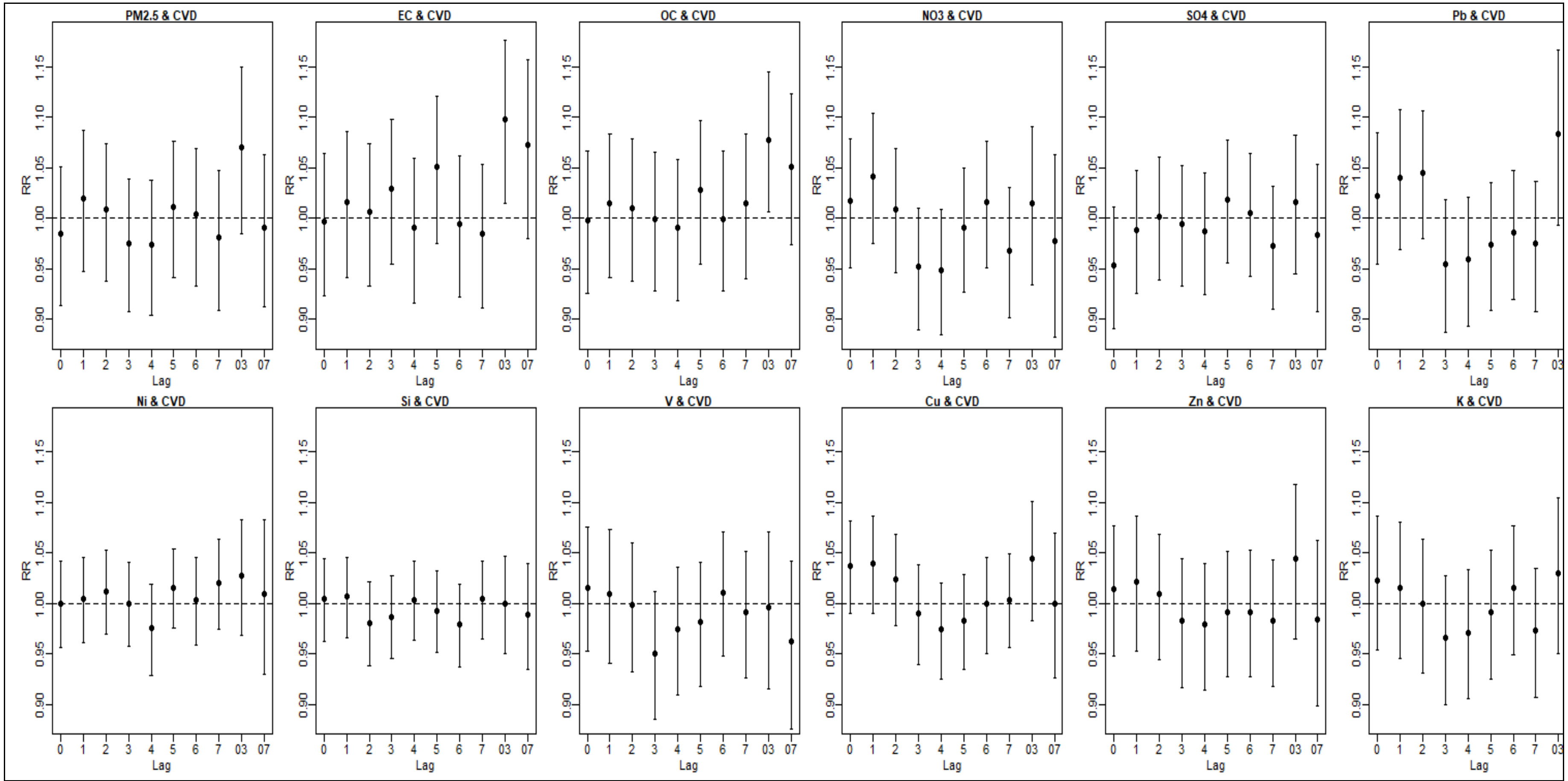
PM2.5 & CVD
EC & CVD
OC & CVD
NO3 & CVD
SO4 & CVD
Pb & CVD
Ni & CVD
Si & CVD
V & CVD
Cu & CVD
Zn & CVD
K & CVD
RR
Lag
0 1 2 3 4 5 6 7 03 07
0.90 0.95 1.00 1.05 1.10 1.15

Daejeon

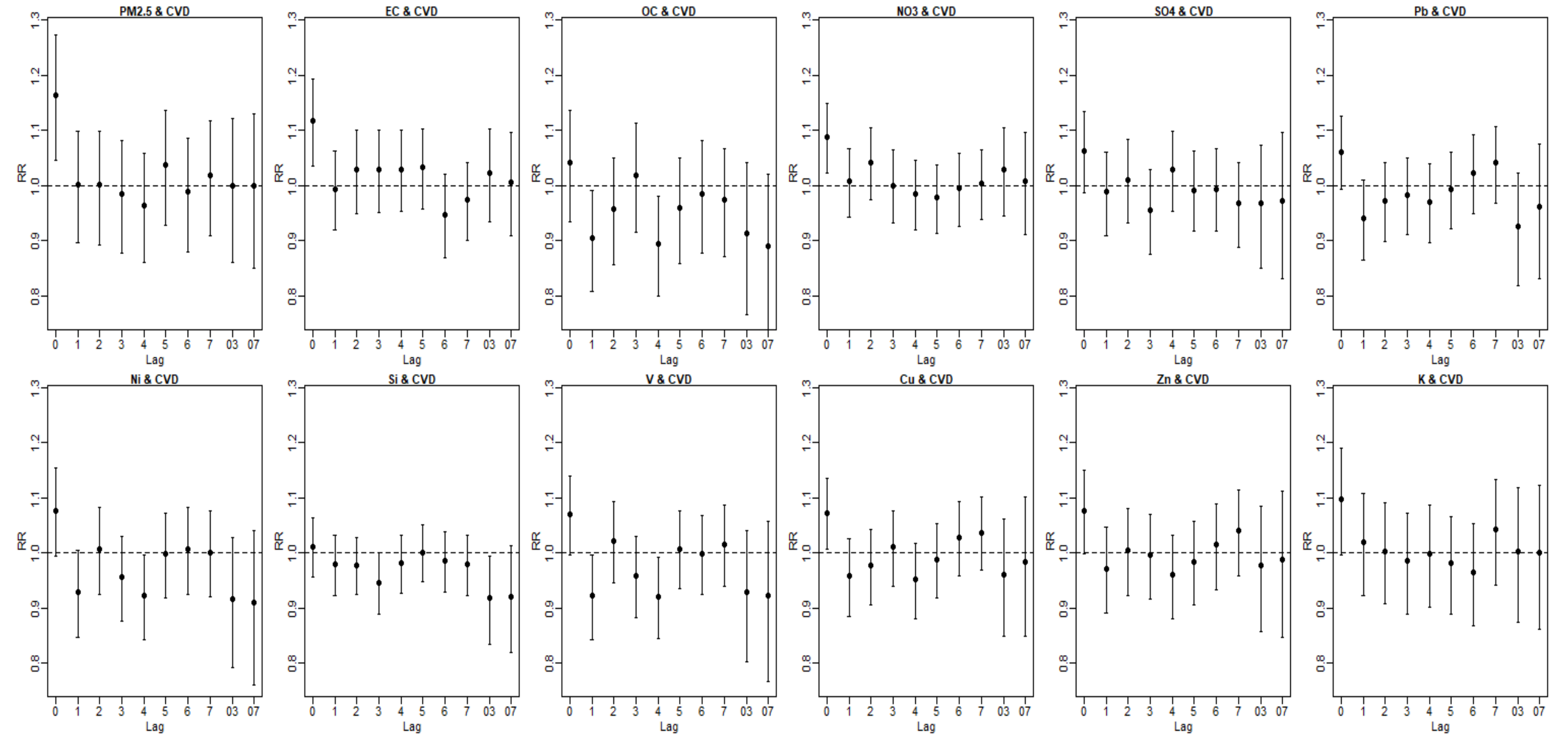

PM2.5 & CVD
EC & CVD
OC & CVD
NO3 & CVD
SO4 & CVD
Pb & CVD
Ni & CVD
Si & CVD
V & CVD
Cu & CVD
Zn & CVD
K & CVD
RR
Lag
0.8
0.9
1.0
1.1
1.2
1.3
0 1 2 3 4 5 6 7 03 07

Ulsan

C. Respiratory mortality

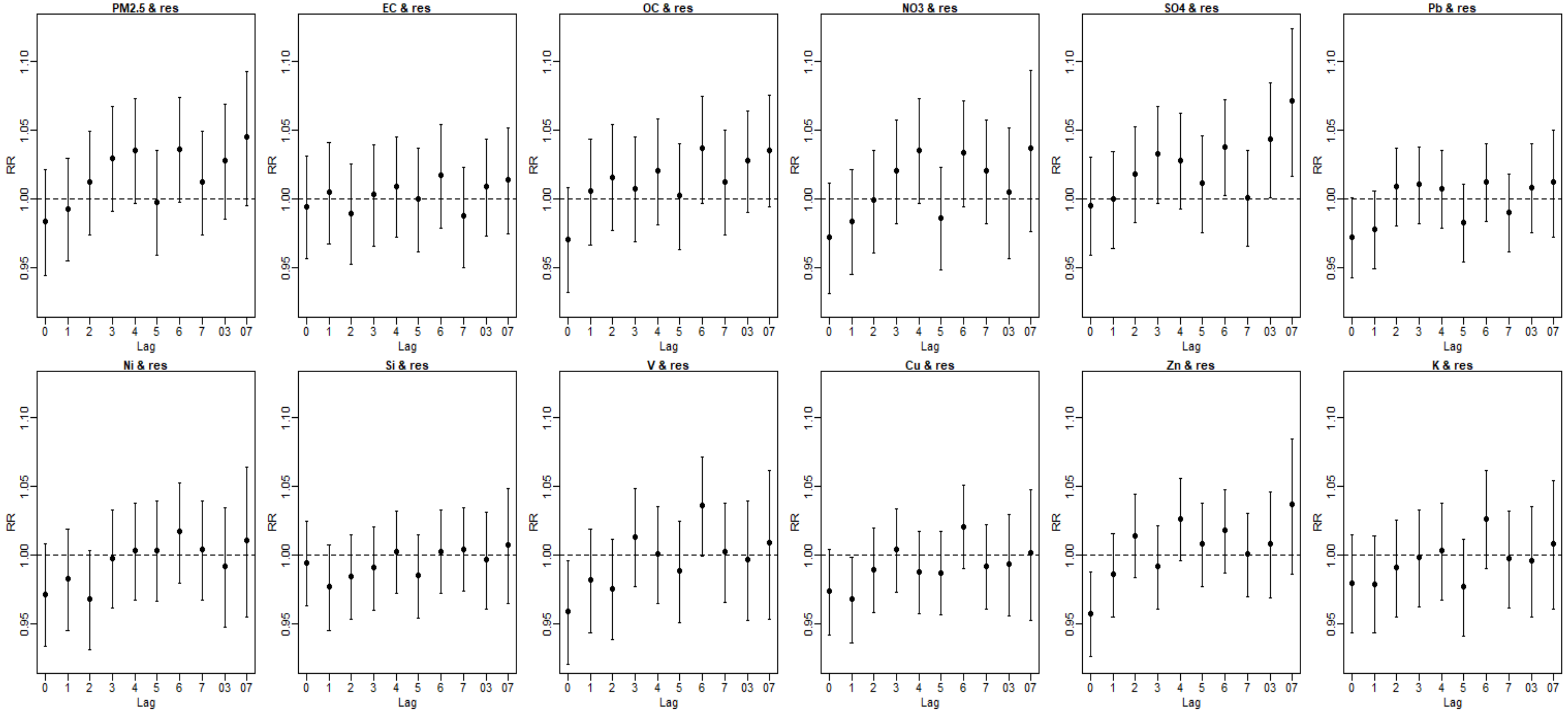

Seoul

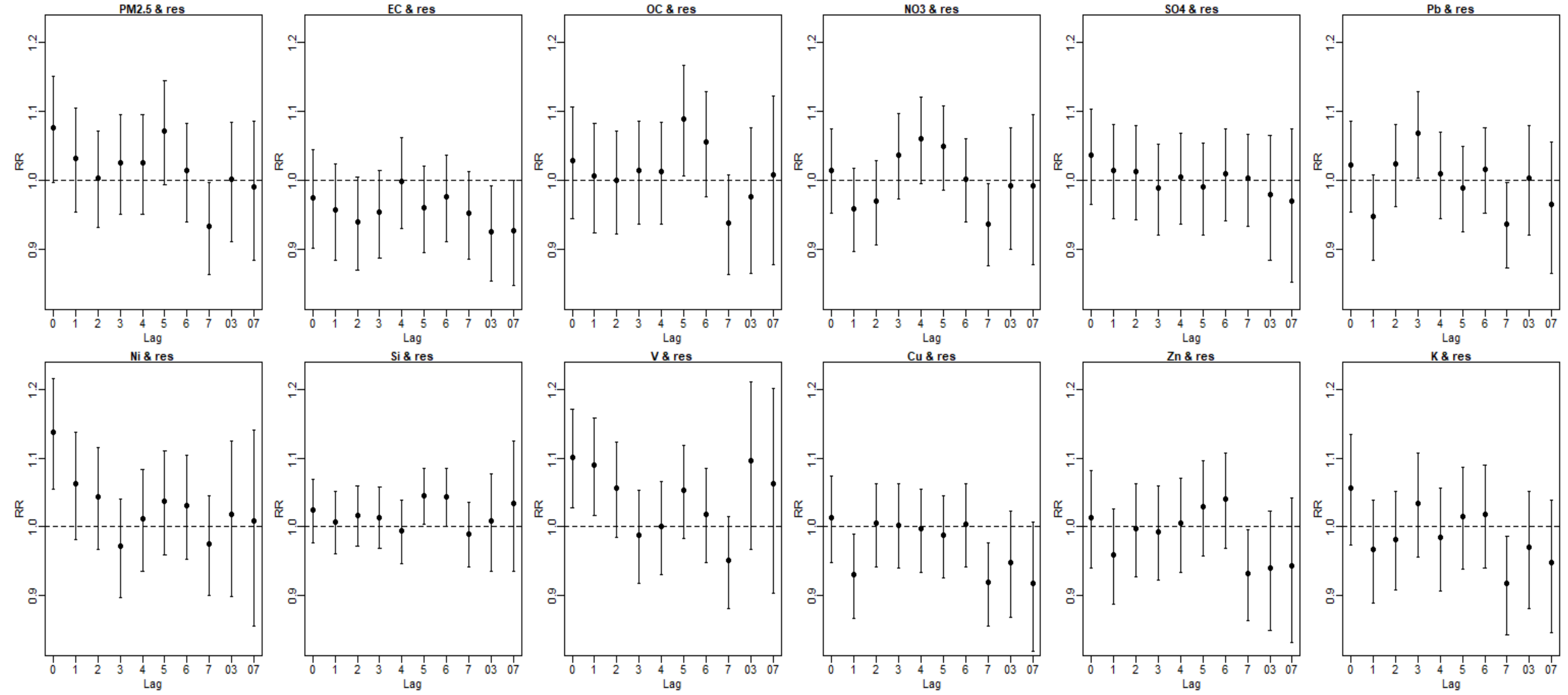
PM2.5 & res
EC & res
OC & res
NO3 & res
SO4 & res
Pb & res
Ni & res
Si & res
V & res
Cu & res
Zn & res
K & res
RR
Lag
0.9
1.0
1.1
1.2
0 1 2 3 4 5 6 7 03 07

Busan

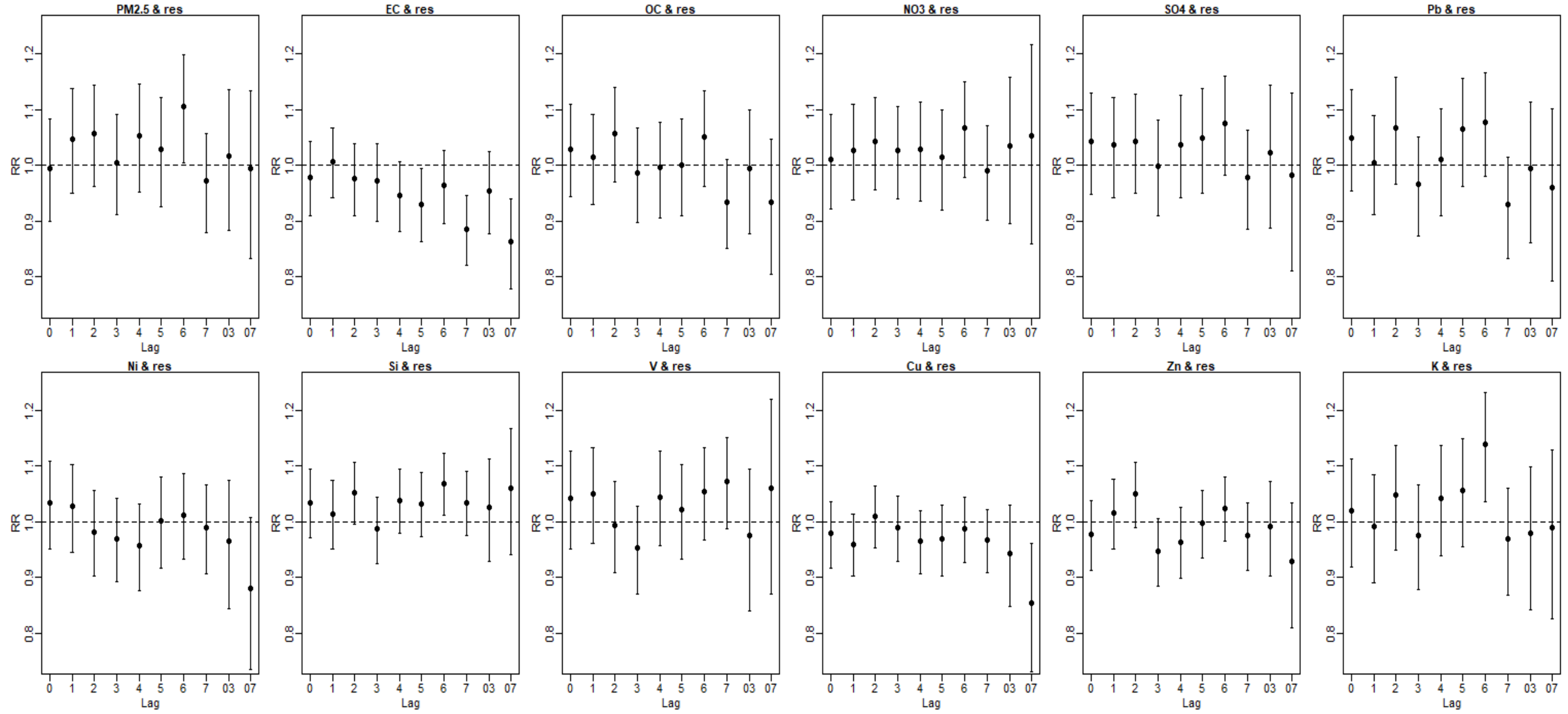
PM2.5 & res
EC & res
OC & res
NO3 & res
SO4 & res
Pb & res
Ni & res
Si & res
V & res
Cu & res
Zn & res
K & res
RR
Lag
0 1 2 3 4 5 6 7 03 07
0.8 0.9 1.0 1.1 1.2

Daegu

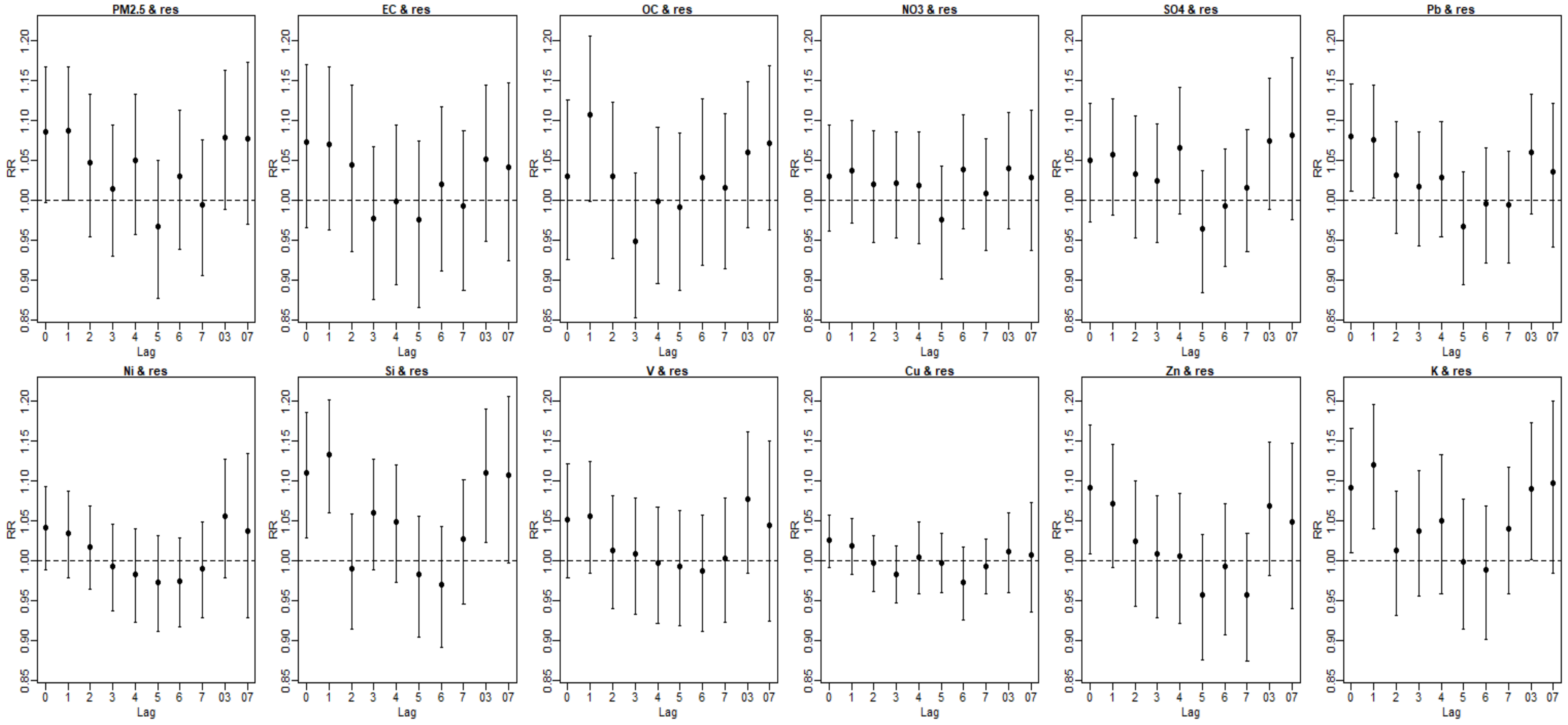
PM2.5 & res
EC & res
OC & res
NO3 & res
SO4 & res
Pb & res
Ni & res
Si & res
V & res
Cu & res
Zn & res
K & res
RR
Lag
0.85
0.90
0.95
1.00
1.05
1.10
1.15
1.20
0 1 2 3 4 5 6 7 03 07

Gwangju

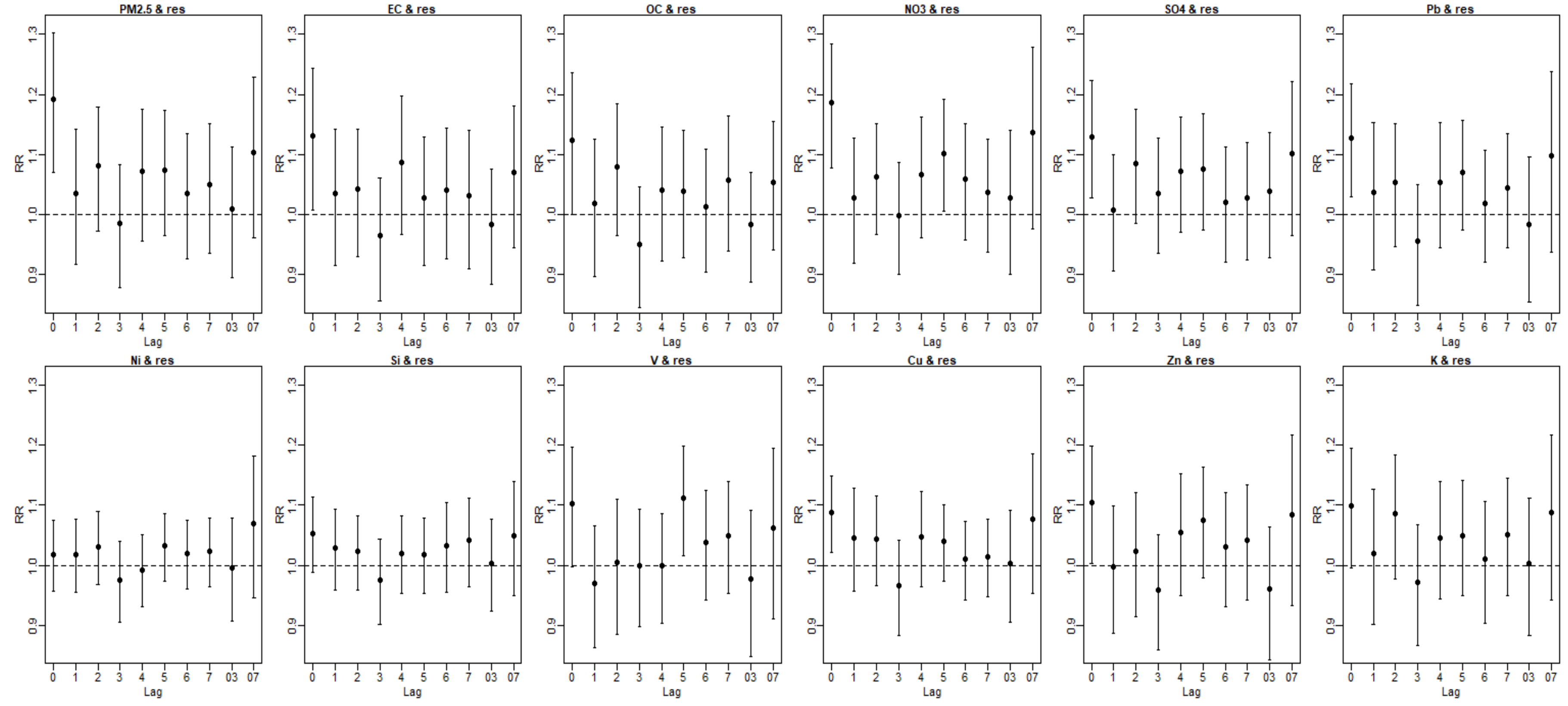
PM2.5 & res
EC & res
OC & res
NO3 & res
SO4 & res
Pb & res
Ni & res
Si & res
V & res
Cu & res
Zn & res
K & res
RR
Lag
0 1 2 3 4 5 6 7 03 07
0.9 1.0 1.1 1.2 1.3

Daejeon

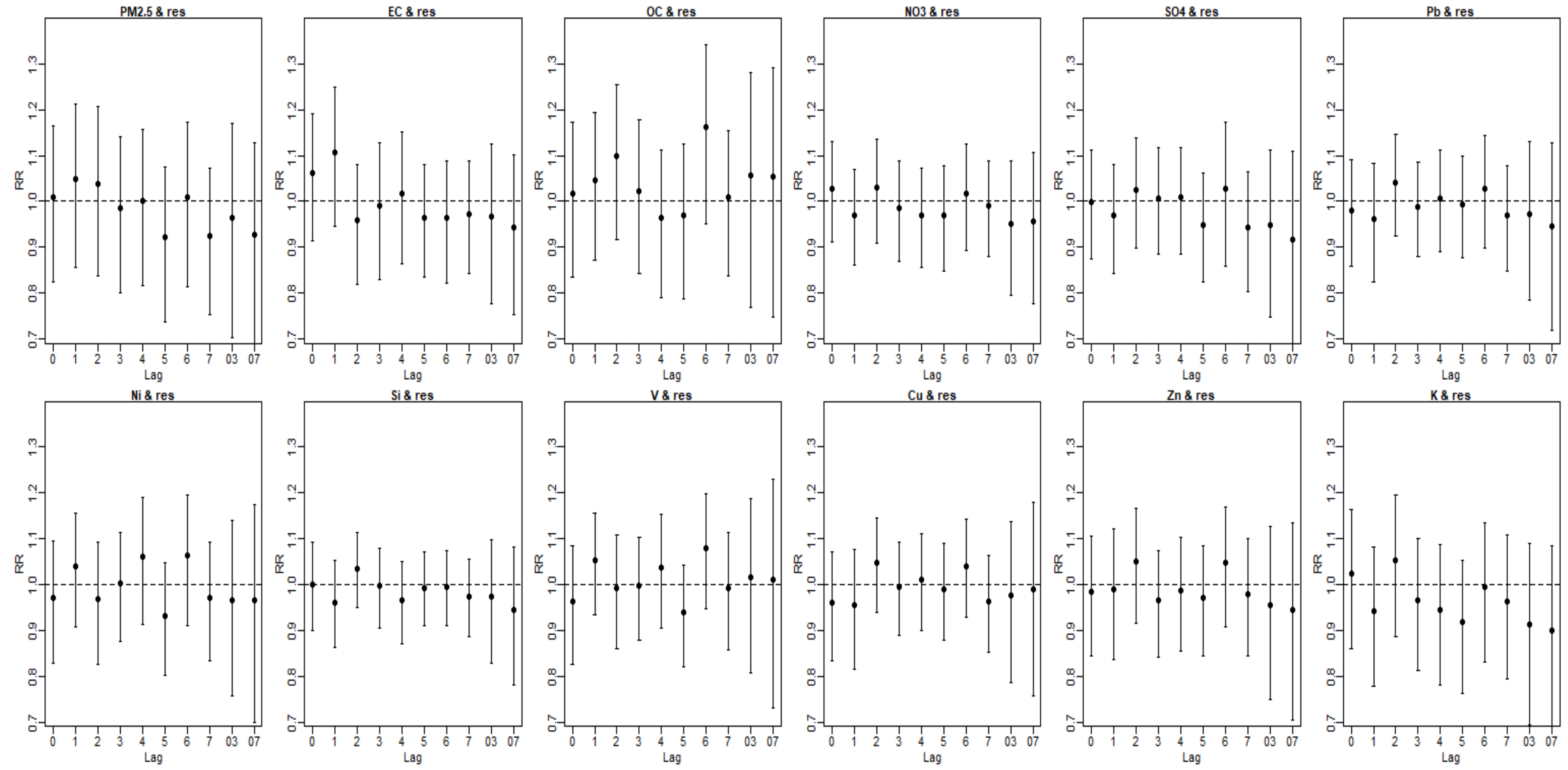

Ulsan

Figure S2. Relative risks (RRs) and 95% confidence intervals of daily mortality for interquartile range increases in daily concentrations of $PM_{2.5}$ and 11 $PM_{2.5}$ components across seven single-day lags (lag 0 to lag 7) and two multiday lags (lag 0–3 and lag 0–7), in each of the six South Korean major cities from 2013 to 2015.

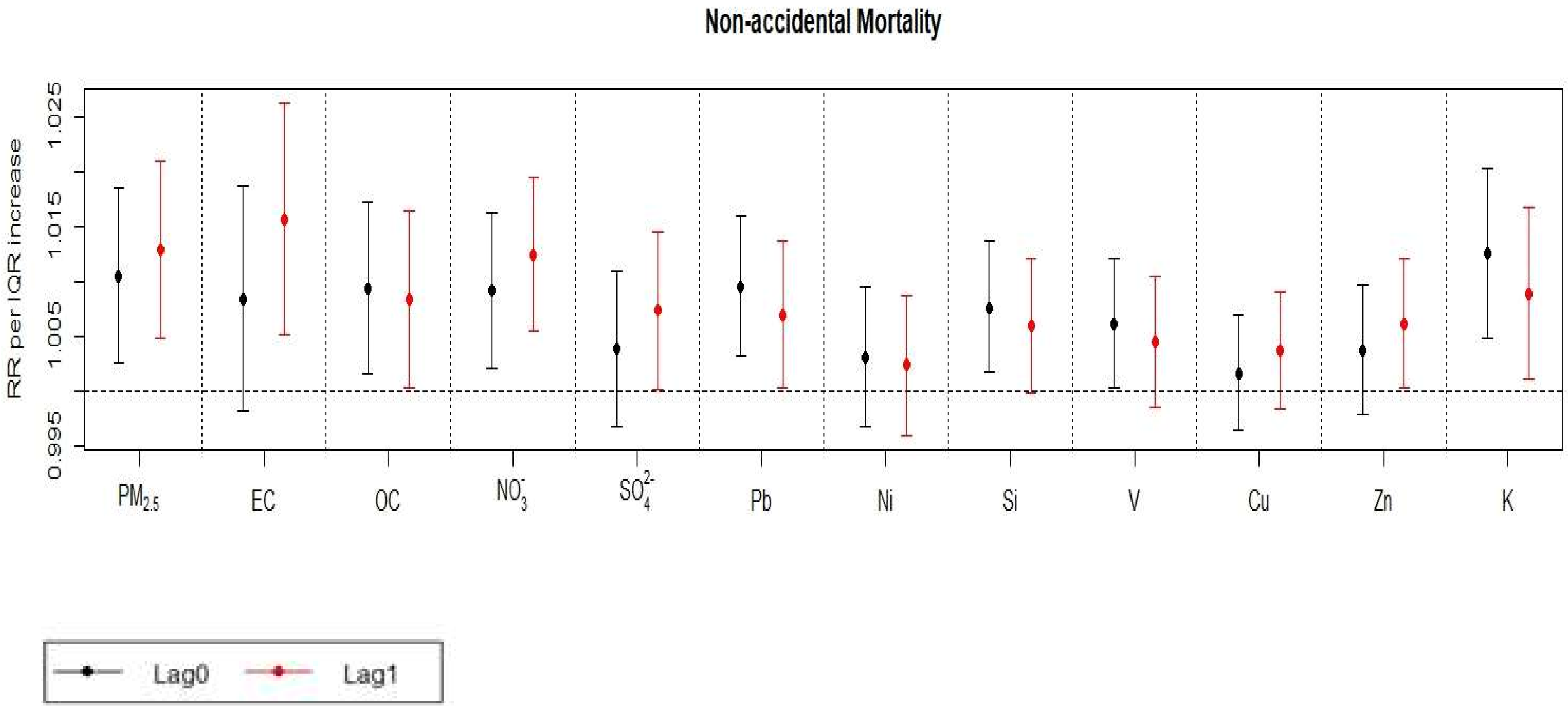

Figure S3. Relative risks (RRs) and 95% confidence intervals of daily nonaccidental mortality for interquartile range increases in daily concentrations of $PM_{2.5}$ and 11 $PM_{2.5}$ chemical components over all six South Korean major cities from 2013 to 2015.

A. Non-accidental mortality

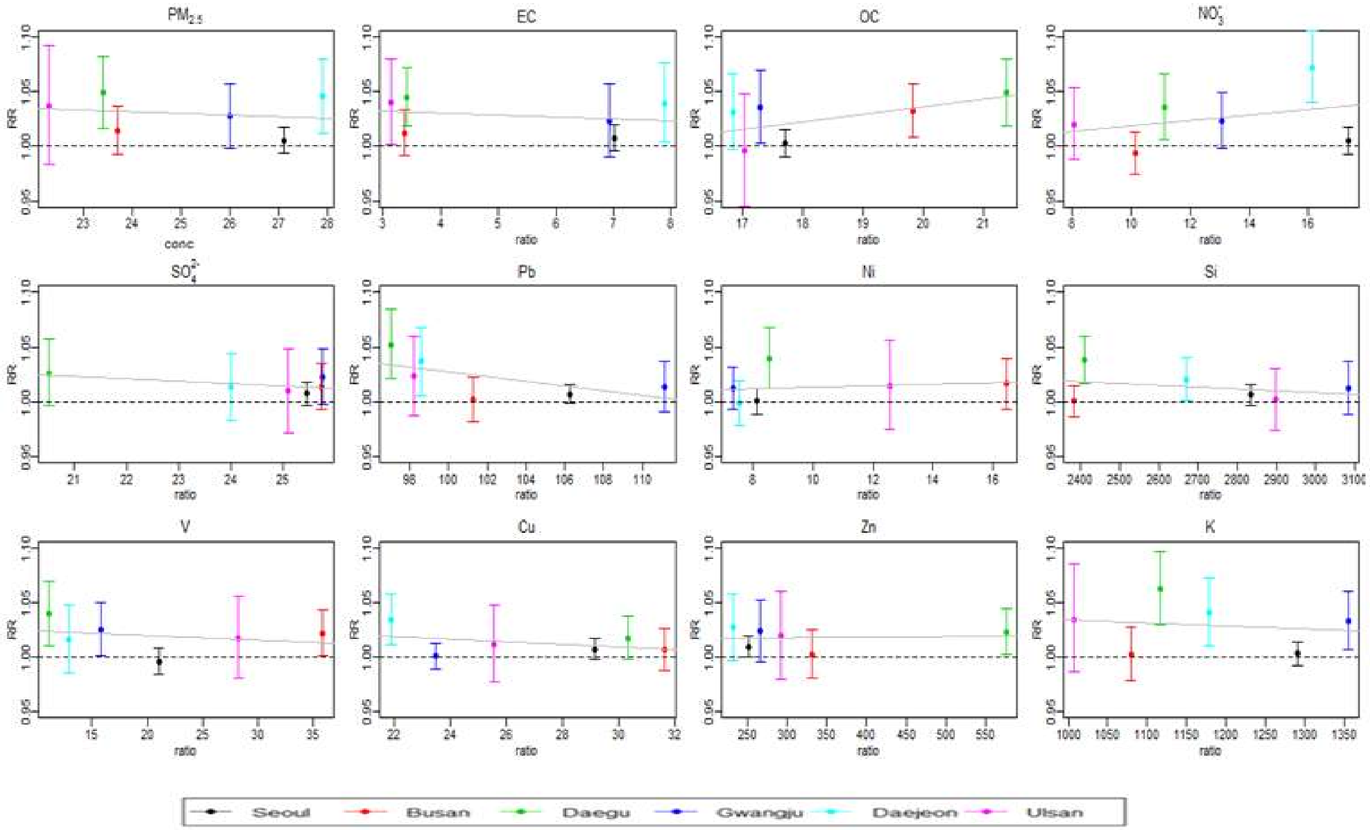

B. Cardiovascular mortality

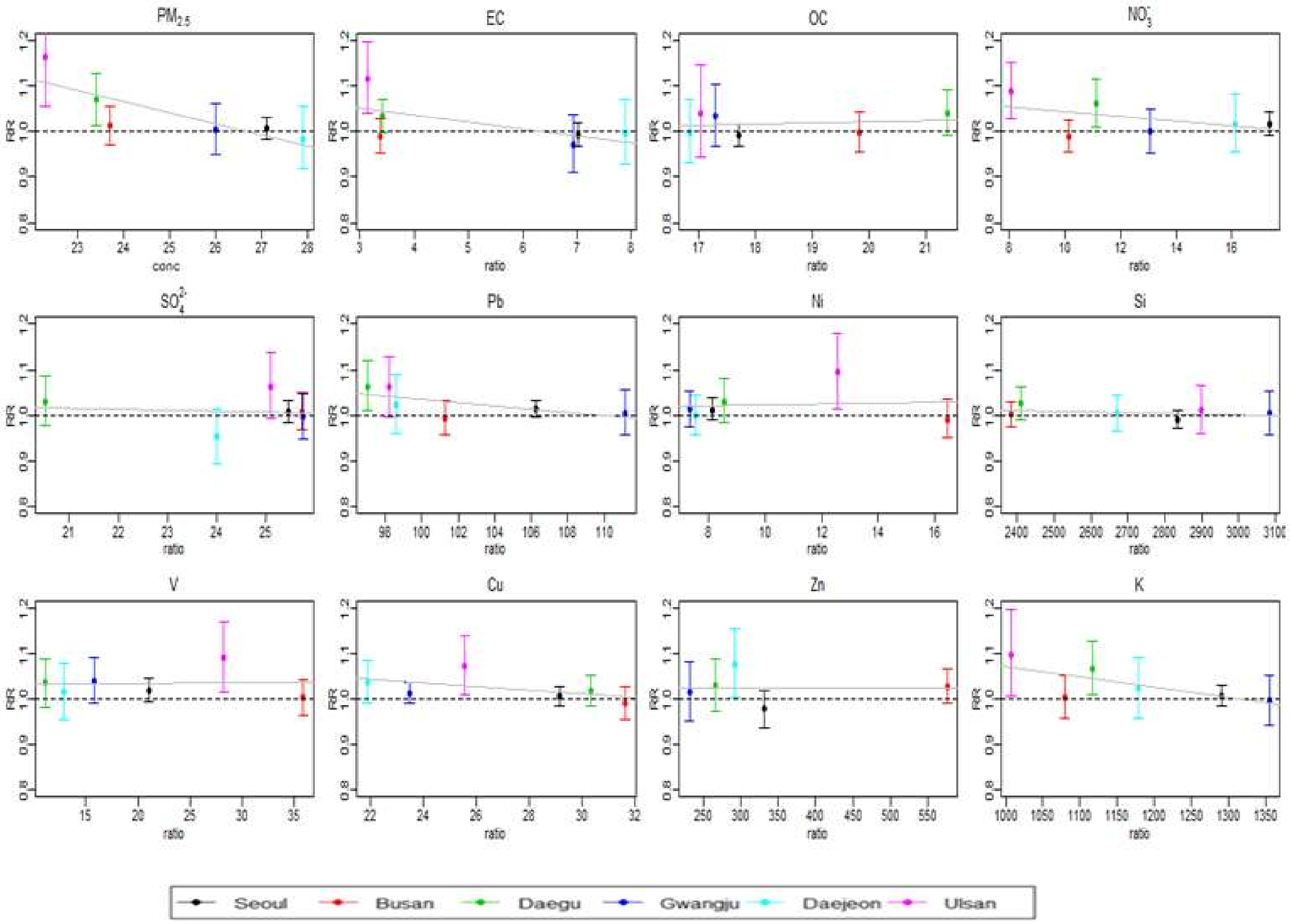

C. Respiratory mortality

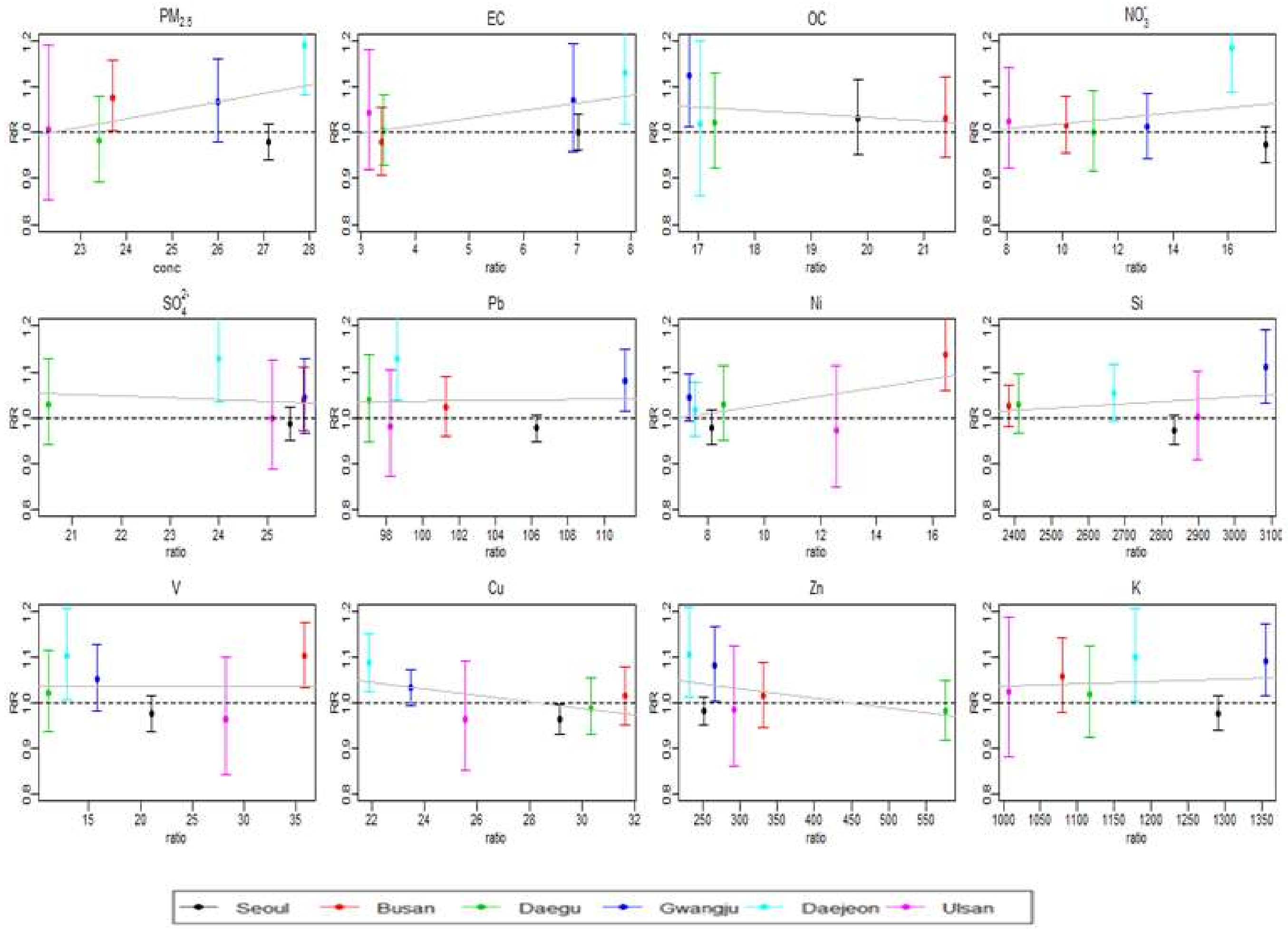

Figure S4. Relative risks (RR)s and 95% confidence intervals (CIs) of mortality for $PM_{2.5}$ components against the ratios of $PM_{2.5}$ component concentrations to $PM_{2.5}$ concentrations across six South Korean major cities from 2013 to 2015 (ratio = 100 X $PM_{2.5}$ component concentration/$PM_{2.5}$ concentration; for $PM_{2.5}$, RRs were plotted against concentrations).

A. Non-accidental mortality

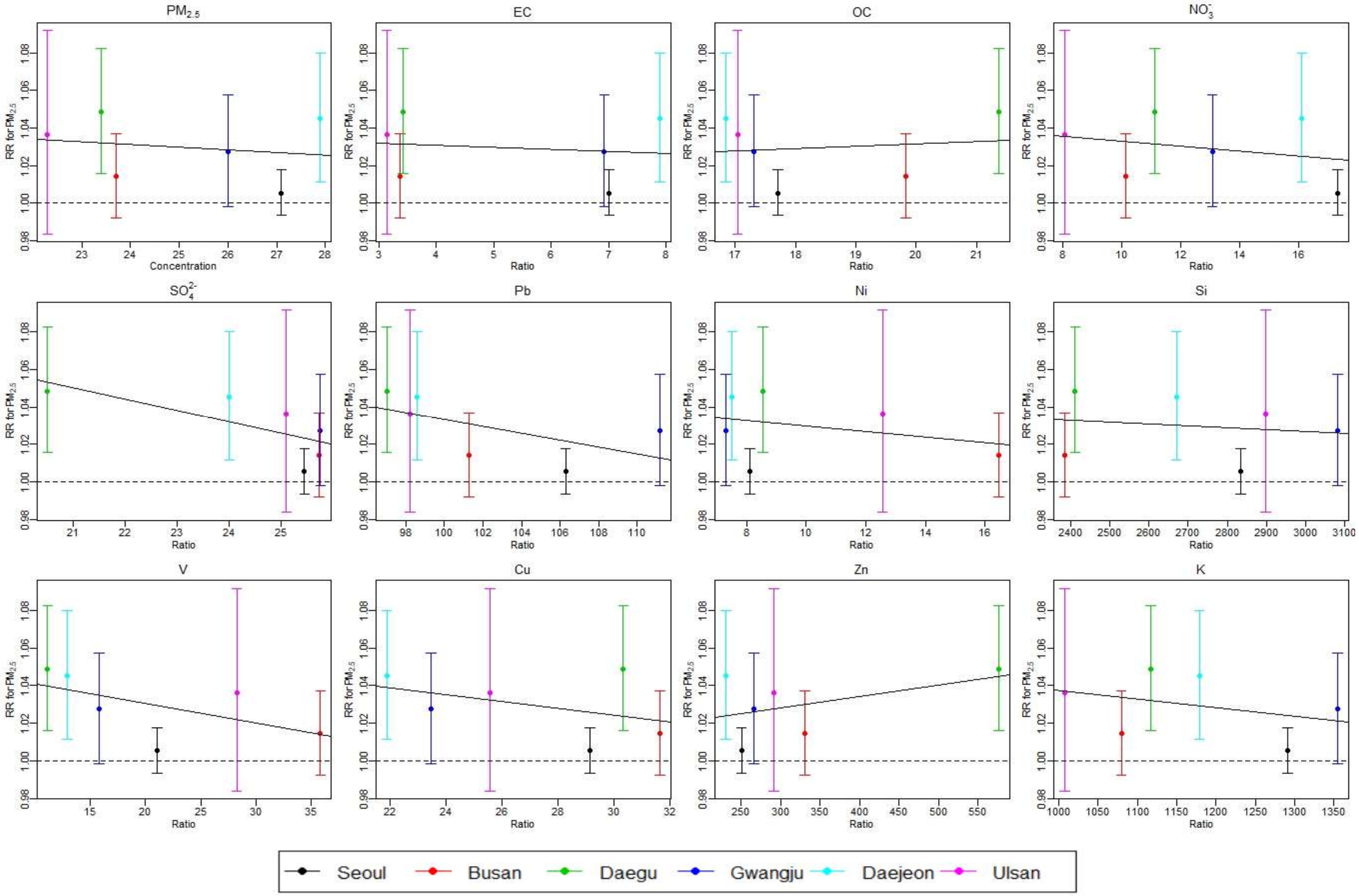

B. Cardiovascular mortality

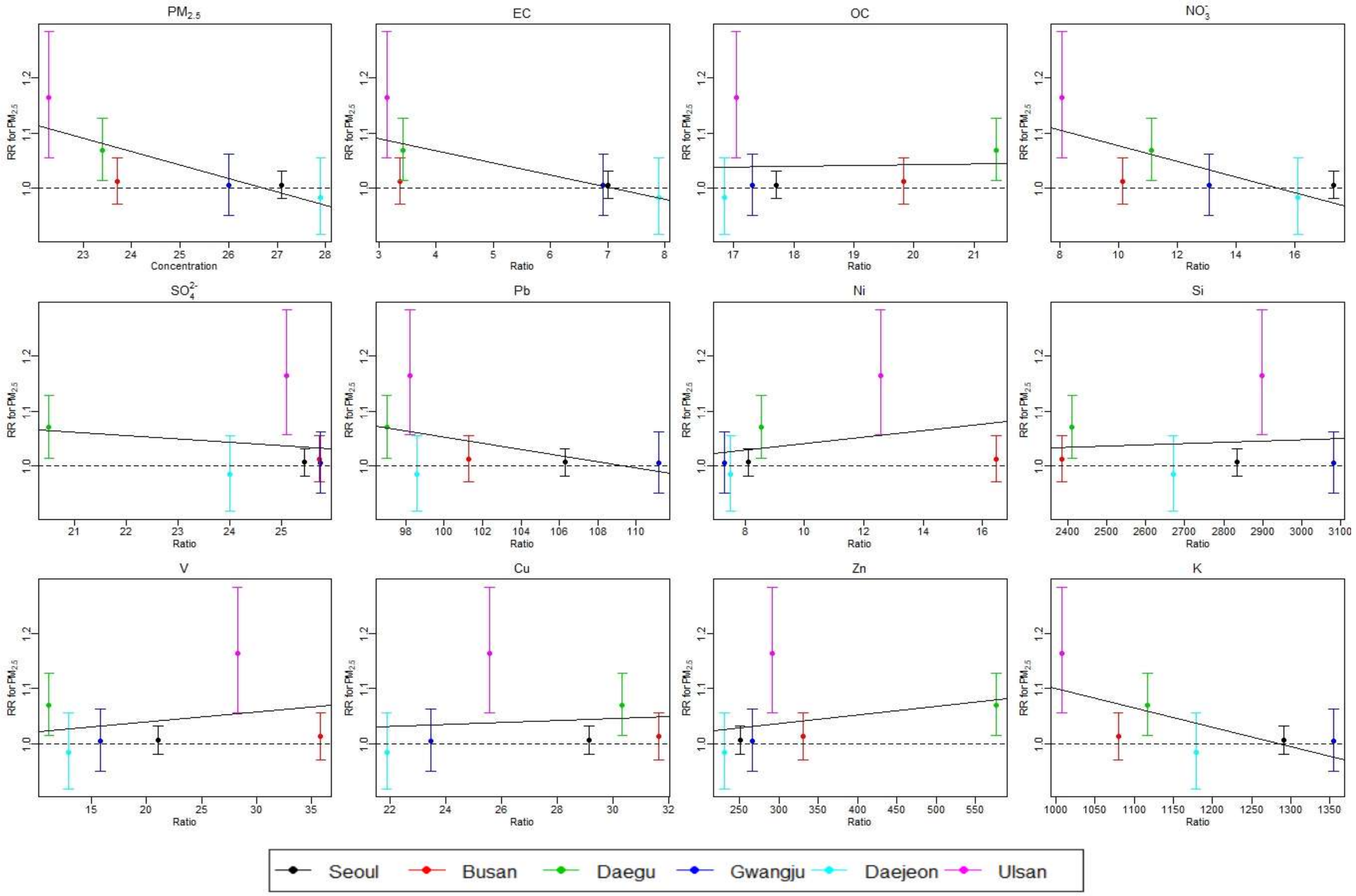

C. Respiratory mortality

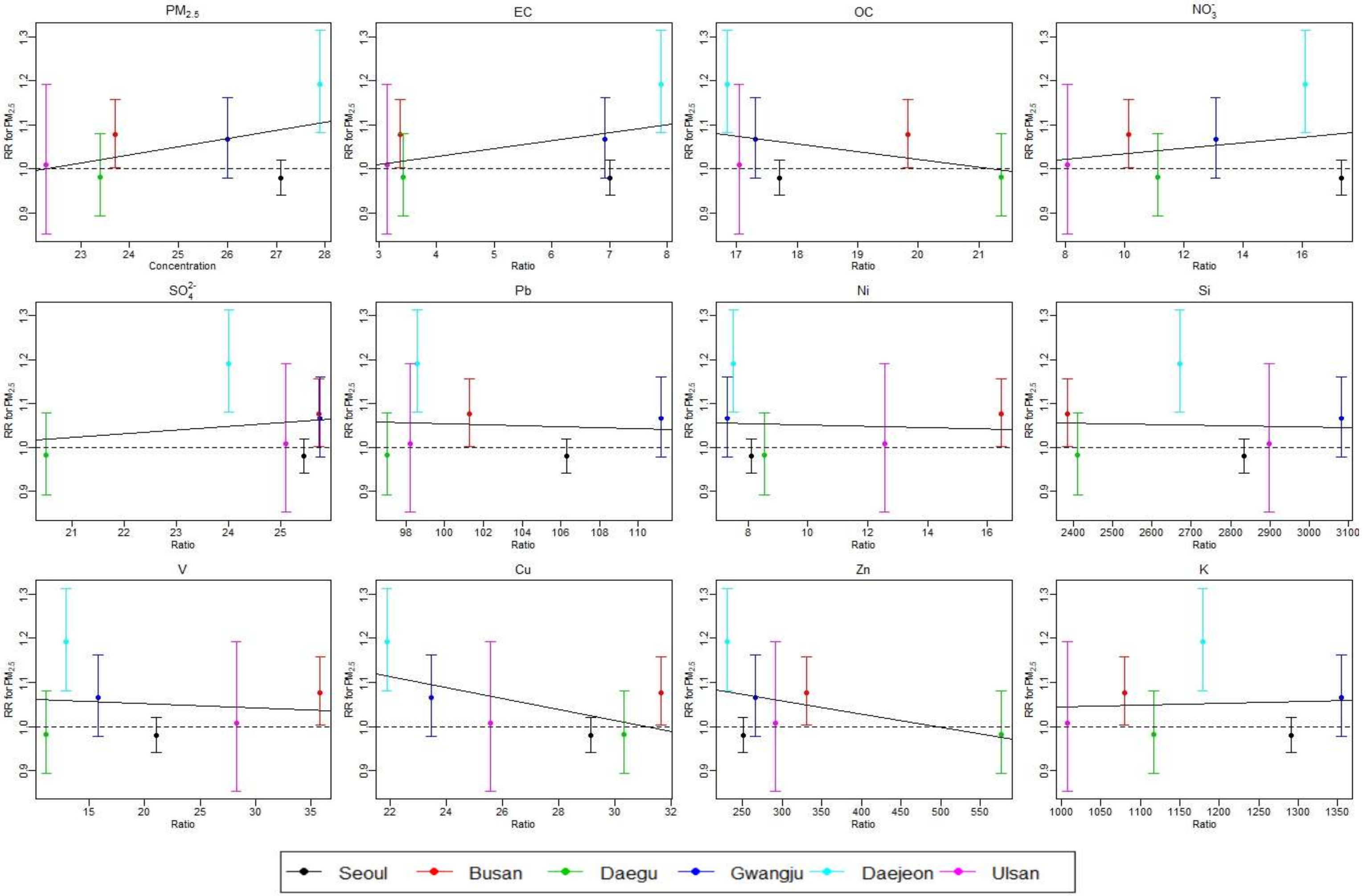

Figure S5. Relative risks (RR)s and 95% confidence intervals (CIs) of nonaccidental mortality for $PM_{2.5}$ against the ratios of $PM_{2.5}$ component concentrations to $PM_{2.5}$ concentrations across six South Korean major cities from 2013 to 2015 (ratio = 100 X $PM_{2.5}$ component concentration/$PM_{2.5}$ concentration; for $PM_{2.5}$, RRs were plotted against concentrations).

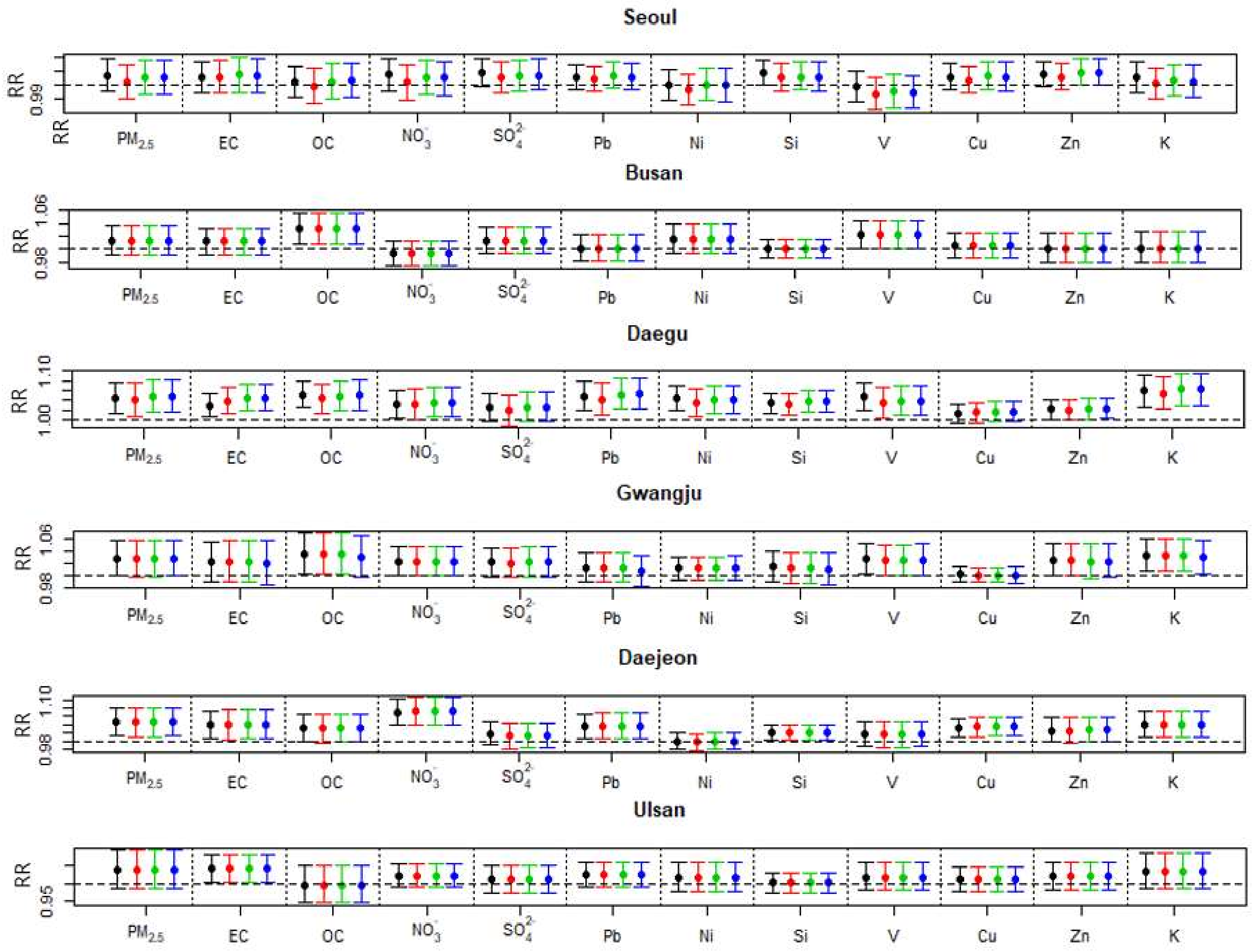

Figure S6. Relative risks (RRs) and 95% confidence intervals of nonaccidental mortality for 11 $PM_{2.5}$ components by different degrees of freedom (df) used for temporal trend adjustment (our primary model with df 6 per year) (black, red, green, green, and blue for df 2, 4, 6, and 12).

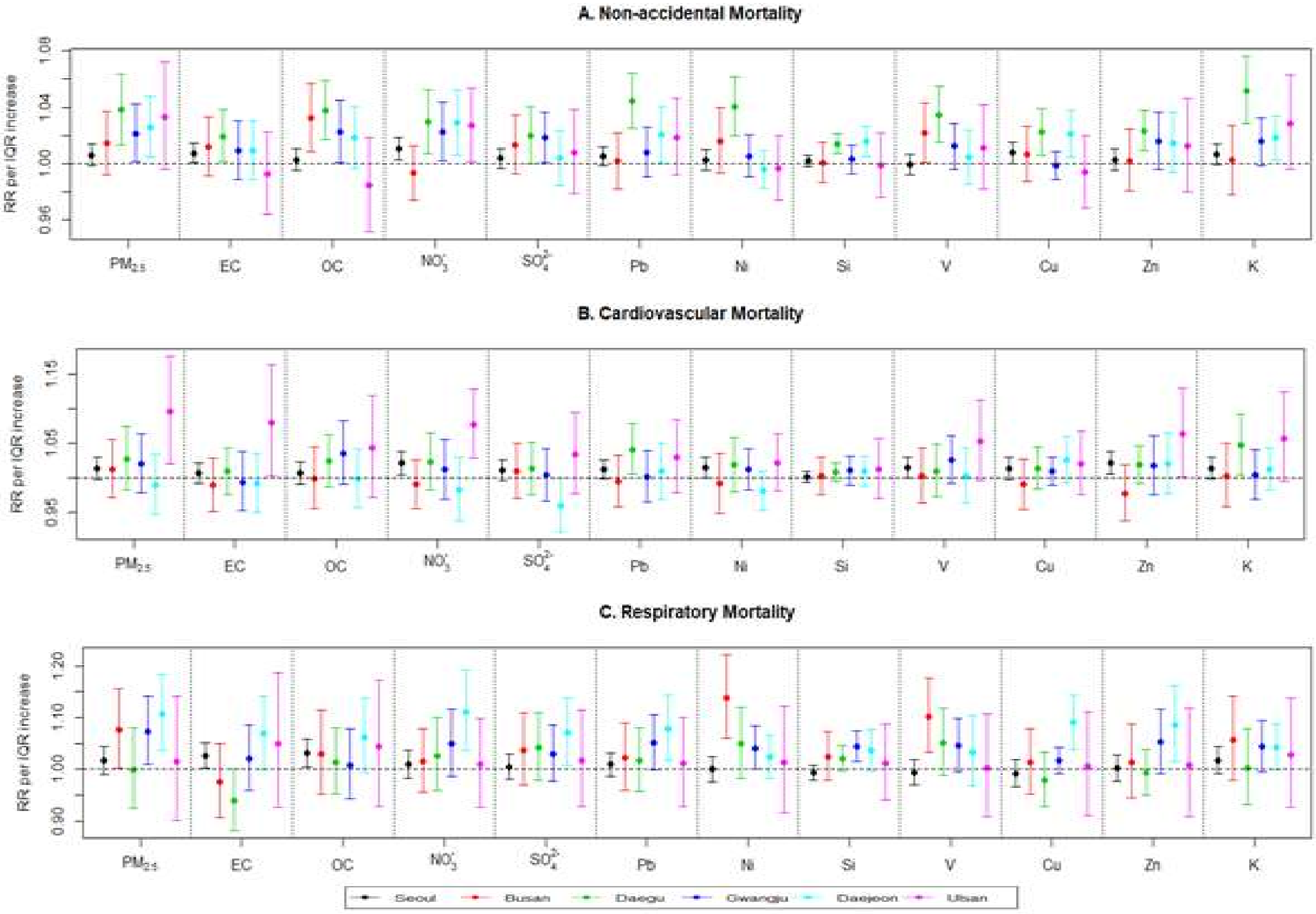

Figure S7. Relative risks (RRs) and 95% confidence intervals of daily mortality for interquartile range increases in daily concentrations of $PM_{2.5}$ and 11 $PM_{2.5}$ components for all available years between 2011 and 2015.
